# Synthesis of the l- and d-SH2 domain of the leukaemia oncogene Bcr-Abl†

**DOI:** 10.1039/d2cb00108j

**Published:** 2022-07-06

**Authors:** Nina Schmidt, Frank Abendroth, Olalla Vázquez, Oliver Hantschel

**Affiliations:** Institute of Physiological Chemistry, University of Marburg 35032 Marburg Germany oliver.hantschel@uni-marburg.de; Faculty of Chemistry, University of Marburg 35032 Marburg Germany olalla.vazquez@staff.uni-marburg.de; Center for Synthetic Microbiology (SYNMIKRO), University of Marburg 35032 Marburg Germany

## Abstract

The d- and l-versions of the Bcr-Abl SH2 domain (12.7 kDa) were synthesized. Key optimizations included pseudoproline incorporation, *N*-terminal hydrophilic tail addition and mild *N*-acetoxy succinimide acetylation. Their folding and activity are as for the recombinant protein. Our results will enable engineering of mirror-image monobody antagonists of the central oncoprotein Bcr-Abl.

## Abstract 2

The d- and l-versions of the Bcr-Abl SH2 domain (12.7 kDa) were synthesized successfully and show similar folding as compared to a recombinantly expressed version.

## Graphical abstract

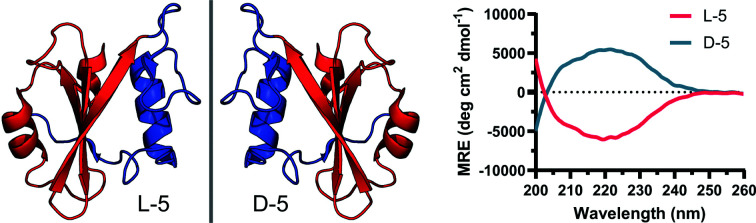

Bcr-Abl is the central driver oncogene of chronic myelogenous leukemia (CML) and subsets of B-cell acute lymphoblastic leukemia.^1^ It is a fusion protein, whose expression results from a chromosomal translocation of the breakpoint cluster region (BCR) and Abelson tyrosine kinase (ABL) genes. Bcr-Abl is a deregulated highly active tyrosine kinase and can be potently inhibited by imatinib (Gleevec), the first molecular targeted cancer drug.^1^ Although imatinib and its successors have strongly improved survival of most CML patients, drug resistance mutations result in disease recurrence.^2^ Our previous work identified alternative approaches to target Bcr-Abl allosterically through disruption of an intramolecular protein–protein interaction of the Src homology 2 (SH2) domain and the kinase domain.^3^ This was achieved by engineered high-affinity monobody proteins, which bound the SH2 domain and inhibited Bcr-Abl activity, downstream signalling and leukemogenesis.^4^

SH2 domains bind tyrosine-phosphorylated peptide sequences with low micromolar affinities and are among the largest families of human protein–protein interaction domains.^5^ SH2 domains are important drug targets, but the development of selective SH2-inhibitors, such as high-affinity peptides,^6^ peptidomimetics or small molecules, is notoriously difficult.^7^ In contrast, we developed various monobodies that bound different SH2 domains with nanomolar affinities and unprecedented selectivity.^3,4,8–11^ The lack of disulfide bonds in the fibronectin type III (FN3) monobody scaffold enables their activity in the reducing environment of the cytoplasm and resulted in inhibition of signalling of the target oncoprotein.^11^ The small size of monobodies (∼10 kDa) could show better tissue penetration and easier intracellular delivery into tumours. On the other hand, the clinical use of monobodies may be hampered by a low plasma half-life and induction of an immune response. A strategy to improve these properties relies on the generation of mirror-image monobodies consisting of d-amino acids. d-Proteins are non-immunogenic, metabolically more stable and have a longer half-life *in vivo*, because their amide bonds are not substrates of proteases.^12,13^ Engineering of d-monobodies requires synthesis of the target protein in d-configuration by solid phase peptide synthesis (SPPS) and native chemical ligation (NCL, Fig. 1). The resulting d-target can be subjected to standard selection with a combinatorial l-monobody library. Once a high-affinity l-monobody is identified, its counterpart is synthesized in d-configuration and refolded, which should consequently bind to the initial l-target. Similar strategies were applied to perform mirror-image phage display or natural product library screenings for the identification of d-peptide, d-protein or mirror-image small molecule binders of various proteins.^12–18^

**Fig. 1:**
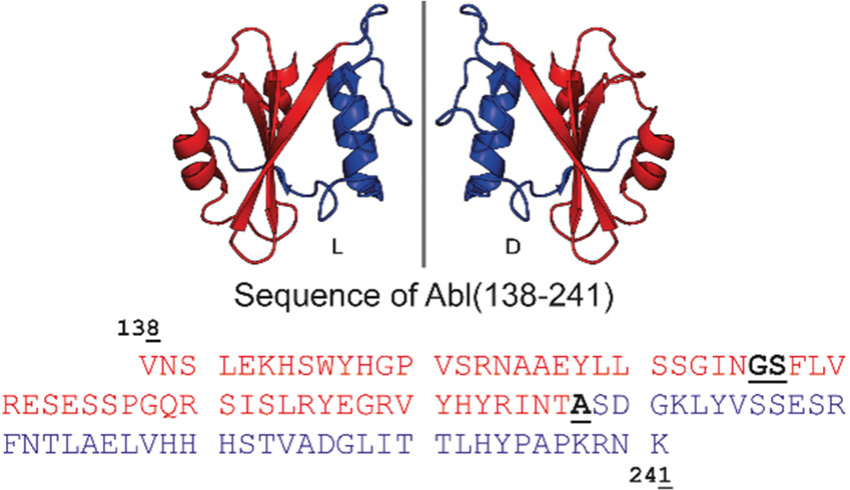
Cartoon representation of the Bcr-Abl SH2 domain, its mirror-image version and corresponding amino acid sequences from V_138_ to K_241_. The *N*- and *C*-terminal parts are in red and blue, respectively. Incorporation of the glycine–serine (GS) pseudoproline dipeptide (Fmoc-Gly-Ser(psiMe,Mepro)-OH) and the ligation site (A) are underlined and highlighted in bold black in the sequence. The sequence numbering is based on Abl isoform 1B. The structure of the Bcr-Abl SH2 domain was obtained from PDB (ID: 3K2M).

Other noteworthy applications of d-proteins include building mirror-image biological systems by synthesis of an enzymatically active d-DNA ligase^19^ or using d-proteins as protein crystallization chaperones.^12,18^

Given the complex allosteric and orthosteric interactions of the Bcr-Abl SH2 domain, synthesis of its full native sequences and correct folding and function of the synthetic d-protein must be assured. Here, we report the efficient synthesis, native conformation and function of the mirror-image version of the Bcr-Abl SH2 domain, which will be used for future mirror-image monobody development by phage- and yeast-display selection.

The large size of the Bcr-Abl SH2 domain (104 aa) hampers its synthetic accessibility by routinely linear Fmoc based-SPPS.^20^ In the past few years, flow chemistry has risen as an efficient approach for producing single-domain proteins of up to 164 aa in one step.^21^ However, the unavailability of commercial synthesizers, low atom economy, material yield as well as increased side reactions at elevated temperatures limit its application.^22^ Consequently, NCL remains the golden standard for chemical protein synthesis.^23,24^ Along these lines, NCL has enabled the synthesis of functionally active proteins up to 467 aa,^25^ the incorporation of post-translational modifications^26^ and production of mirror-image versions,^12^ which is not possible by recombinant protein expression techniques.^20^ Importantly, the vast developed methodology including chemical auxiliaries,^27–29^ desulfurization reactions,^30,31^ diselenide–selenoester ligations (DSL)^32^ and others^33–35^ enables protein synthesis beyond cysteine-containing ones.^36^ Since the Bcr-Abl SH2 domain lacks native cysteines, we envisioned a convergent two-segment synthesis where T_197_–A_198_ is the ligation junction (Fig. 1), and consequently, relying on the post-ligation desulfurization concept of Dawson.^30^ The result of this design is the splitting of the SH2 domain into the *N*- and *C*-terminal fragments Abl1(138–197) (1) and Abl1(198–241) (3), respectively (Fig. 2 and Fig. S1, ESI†). In the latter, the *N*-terminal alanine is mutated to cysteine. Synthesis was first attempted and optimized for the l-Bcr-Abl SH2 domain before the d-counterpart was synthesized.

**Fig. 2:**
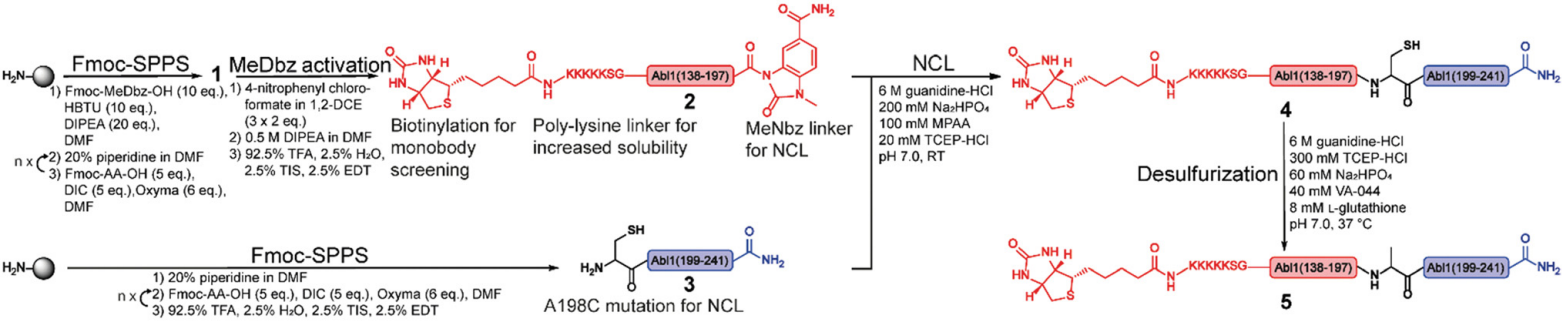
Synthetic strategy for the synthesis of the Bcr-Abl SH2 domain. The N- and C-terminal fragments are in red and blue, respectively, and are synthesized *via* solid phase peptide synthesis (SPPS). The N-terminal peptide 1 corresponds to the biotinylated Bcr-Abl SH2 domain (138–197) with C-terminal MeDbz linker, which is activated to MeNbz on resin. After cleavage from the resin, the resulting peptide 2 can undergo native chemical ligation (NCL) with the cysteine (Cys)-containing peptide 3 to yield polypeptide 4. Subsequent desulfurization of this Cys variant of Bcr-Abl SH2 (4) provides the final SH2 domain (5). MeDbz: 3-amino-4-(methylamino)benzoic acid; MeNbz: *N*-acyl-*N*′-methylacylurea; NCL: native chemical ligation.

For the generation of the *C*-terminal thioester of the *N*-terminal segment 1, we used the 3-amino-4-(methylamino)benzoic acid (MeDbz) linker due to commercial availability, suppressed branching during amino acid couplings and mild activation procedure.^37^ SPPS of the *C*-terminal segment Abl1(198–241) (3) was straightforward (12% yield) (Fig. S8 and S9, ESI†). In contrast, solubility problems were encountered in the *N*-terminal fragment Abl1(138–197) (1), and expected to worsen when biotin was coupled, which is required for target immobilisation during monobody selection. To overcome this issue, the addition of five lysine residues (K5) and a serine–glycine linker (SG) to the *N*-terminus resulted in great improvement of solubility (up to mM). Analysis of this *N*-terminal fragment after cleavage from resin revealed two truncations between I_164_ and N_165_ (3898.0 Da) and N_165_ and G_166_ (3783.9 Da). Gratifyingly, the incorporation of the corresponding glycine–serine (GS) pseudoproline (Fmoc-Gly-Ser(psiMe,Mepro)-OH, PP) dipeptide^38^ at residues G_166_ and S_167_, and a gentle increase of the synthesis temperature to 50 °C completely suppressed the truncations (Fig. 3A–C, Fig. S2 and S3, ESI†). Interestingly, after the synthesis of 1, which harbours the *C*-terminal MeDbz linker, we detected a mass corresponding to 8056.7 Da (+42 Da), and hypothesized a possible acetylation on the secondary amine of the MeDbz linker because of the standard capping conditions (acetic anhydride/2,6-lutidine/DMF (5 : 15 : 80, v/v); Fig. 3D–G and Fig. S4, ESI†). Indeed, the same issue was observed during the synthesis of a *C. elegans* neuropeptide, where 76% of the MeDbz linker was acetylated by acetic anhydride.^39^ Besides, capping with acetic anhydride after every coupling step is generally not recommended with the MeDbz linker as it prevents the subsequent *N*-acyl-*N*′-methylacylurea (MeNbz) formation and NCL.^40^ However, removing this step could affect crude purity and aggravate peptide purification. Nevertheless, we successfully circumvented the formation of the acetylated side product without resorting to the removal of the capping step during synthesis by exchanging the acetic anhydride for the mild acetylation reagent with preference for primary amines, *N*-acetoxy succinimide (Fig. 3H–I and Fig. S5, ESI†).^41–43^ Afterwards, by sequential treatment with 4-nitrophenyl chloroformate in 1,2-dichloroethane (DCE) and DIPEA in anhydrous DMF the MeDbz linker of peptide 1 is converted to MeNbz (2). This acts as an efficient thioester precursor for the subsequent NCL reaction.^37^ Finally, peptide 2 was cleaved from the resin, HPLC purified and obtained with a yield of 10% (Fig. S6 and S7, ESI†).

**Fig. 3:**
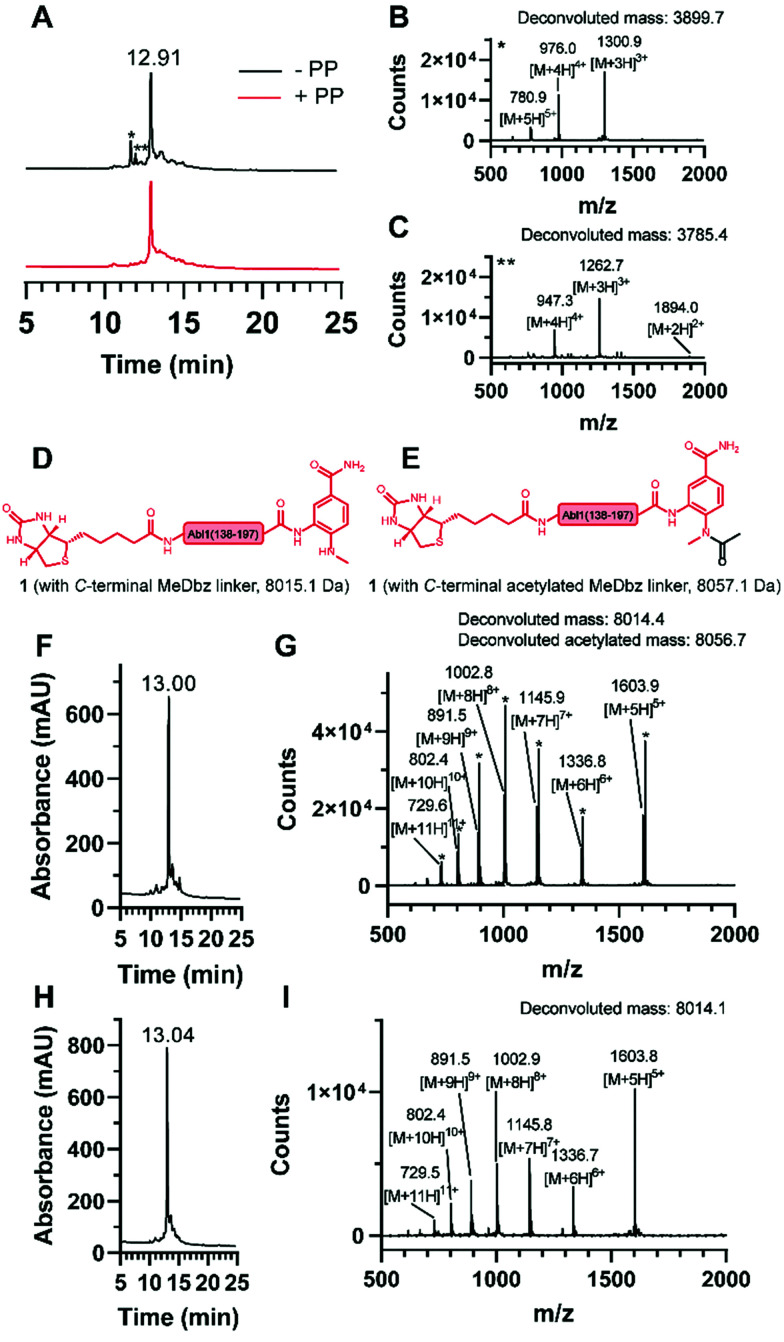
Synthesis of the *N*-terminal fragment (1). (A) Overlay of the chromatograms of the synthesis without (black) and with (red) pseudoproline (PP) incorporation and ESI mass spectrum of the observed truncations (B) * and (C) **. Structures of 1 (D) without and (E) with acetylated MeDbz linker and corresponding molecular weights. (F) Chromatogram and (G) ESI mass spectrum of the synthesis with the capping solution containing acetic anhydride and observation of the MeDbz acetylation. (H) Chromatogram and (I) ESI mass spectrum of the synthesis with the capping solution containing *N*-acetoxy succinimide and suppression of the MeDbz acetylation.

Next, the ligation between both fragments (Fig. 4A and B) was performed at a final concentration of 2 mM in presence of 20 mM TCEP and 100 mM 4-mercaptophenylacetic acid (MPAA) to undergo thiol exchange with MeNbz and generate the corresponding peptide-thioester.^37^ The progress of the ligation was observed *via* HPLC-MS analysis, and typically reached completion after 23 h. Side products only stemmed from the expected competitive hydrolysis of 2 and unreacted 3. After HPLC purification, the ligated polypeptide 4 was obtained with a yield of 51% (Fig. S10 and S11, ESI†).

**Fig. 4:**
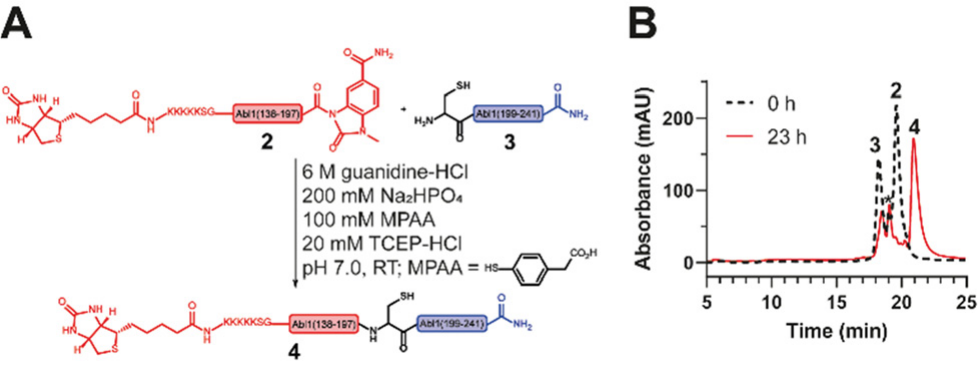
Native chemical ligation (NCL) to obtain the synthetic Bcr-Abl SH2 domain. (A) Scheme of the NCL between the *N*- (2) and *C*-terminal (3) Bcr-Abl SH2 peptides to ligation product 4. (B) Overlay of the chromatograms before (black) and after (red) ligation. A depletion of peptides 2 and 3 is visible while the ligation product 4 emerges. The peak marked with * corresponds to the hydrolysis product of peptide 2.

Subsequently, the cysteine-containing SH2 domain was chemo-selectively desulfurized into the native alanine variant *via* a free-radical-mediated reaction using the radical initiator 2,2′-azobis-[2-(2-imidazolin-2-yl)-propane] dihydrochloride (VA-044).^31^ Nearly quantitative conversion was observed after 16 h at 37 °C. Thus, the purified full length Bcr-Abl SH2 domain (5) was obtained with a yield of 72% (Fig. S12, ESI†) and an overall yield of 4%. Following the same strategy using the d-PP (Fmoc-Gly-d-Ser(psiMe,Mepro)-OH, Fig. S14–S17, ESI†) without further rounds of optimization, a comparable overall yield (5%) was obtained for the d-Bcr-Abl SH2 counterpart (d-5) (Fig. S13, ESI†).

Next, we turned our attention to the protein conformation: the lyophilized synthetic polypeptides were solubilized in 6 M guanidine hydrochloride and a refolding protocol *via* dialysis was optimized (ESI†). Gel electrophoresis analysis revealed that due to the smaller size the recombinant protein migrated slightly lower in the gel compared to the synthetic proteins, which are additionally biotinylated and contain the KKKKKSG linker (Fig. 5A). Size exclusion chromatography analysis showed elution as a single main peak of the synthetic d- and l-Bcr-Abl SH2 domains (Fig. 5B). The elution volume was the same for a recombinantly expressed Bcr-Abl SH2 domain (Fig. S18, ESI†) and corresponded to its monomeric molecular weight. We subsequently recorded far-UV circular dichroism spectra. The recombinant and synthetic l-Bcr-Abl SH2 domains showed very similar spectra in terms of shape, signal strength and depth of minima, in line with a mixed α-helix and β-sheet protein. The synthetic d-Bcr-Abl SH2 domain showed a spectrum mirrored at the *x*-axis compared to the synthetic l-version (Fig. 5C and Fig. S21, ESI†). Contributions of the different secondary structure elements were calculated based on our spectral data showing comparable secondary structure content of all three proteins. These results confirmed that the refolded synthetic proteins had a similar fold as the solubly expressed recombinant protein (Table S6, ESI†).

**Fig. 5:**
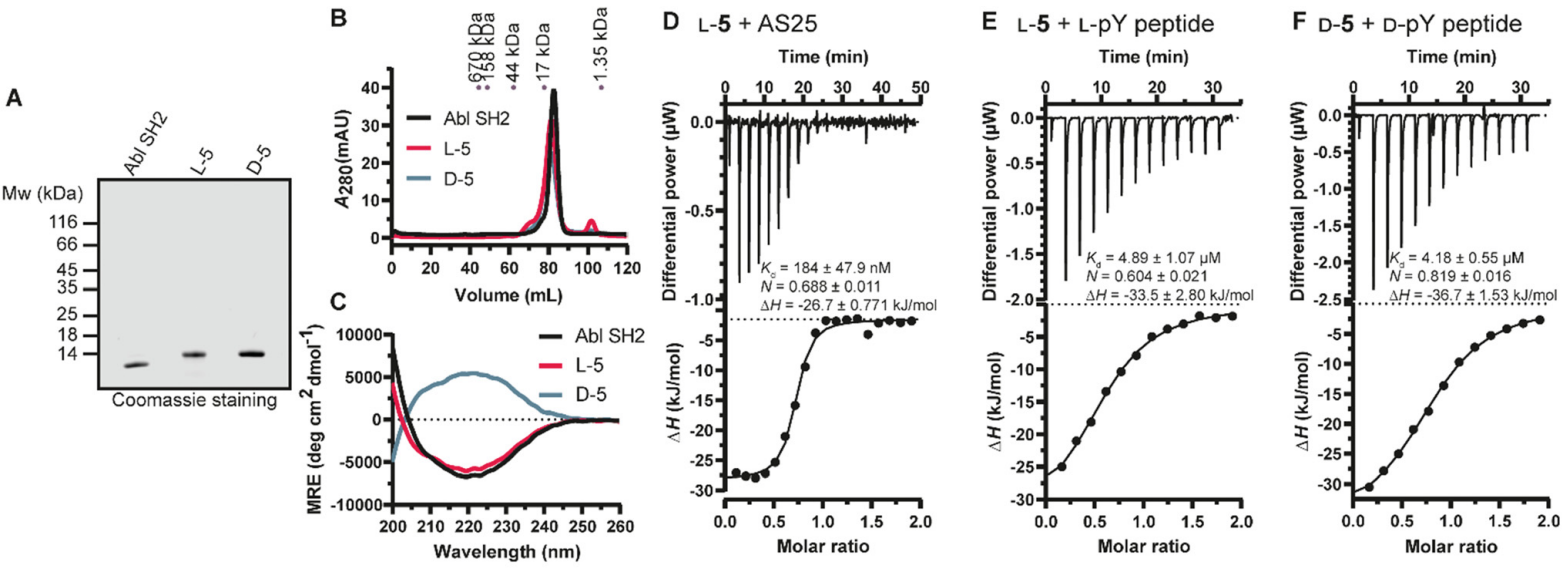
Biochemical characterization of the synthetic Bcr-Abl SH2 domains. (A) Representative sodium dodecyl sulfate–polyacrylamide gel electrophoresis (SDS-PAGE) analysis of recombinant and synthetic l- and d-Bcr-Abl SH2 domains. 5 μg was loaded for each protein. (B) Size exclusion chromatography (SEC) analysis of the final proteins was performed on a 120 mL HiLoad 16/600 Superdex 75 pg column. A calibration of the column with standard proteins is shown on top. (C) Averaged far-UV circular dichroism (CD) spectra of recombinantly expressed and synthetic l- and d-Bcr-Abl SH2 domains. Mean residue ellipticity (MRE) was calculated from two independent measurements. (D–F) Isothermal titration calorimetry (ITC) measurements of synthetic Bcr-Abl SH2 domains with their ligands AS25 and the pYEEI peptide at 25 °C. Each panel shows the raw heat signal of an ITC experiment (top) and the integrated calorimetric data of the area of each peak (bottom). The continuous line represents the best fit of the data based on a 1 : 1 binding model computed from the MicroCal software. A representative measurement of two independent experiments is shown for each example with *K*_d_ value, stoichiometry (*N*) and enthalpy (Δ*H*) calculated from the fit. A list of all obtained *K*_d_ values can be found in Table S4 and S5 (ESI†). (D) AS25 (200 μM) titrated to l-5 (20 μM). (E) l-pY peptide (300 μM) titrated to l-5 (30 μM). (F) d-pY peptide (300 μM) titrated to d-5 (30 μM). The full sequence of the pY peptide is 5 Carboxyfluoresceine-EPQpYEEIPIYLK-CONH_2_.

Thermodynamic stability was assessed by differential scanning fluorimetry. Cooperative unfolding with a melting temperature (*T*_m_) of ∼60 °C for the recombinant and a mildly higher *T*_m_ for the synthetic l- and d-Bcr-Abl SH2 domains were observed (Fig. S22 and Table S7, ESI†).

We next determined whether the synthetic l-protein was capable of binding to the previously extensively characterized monobody targeting the Bcr-Abl SH2 domain, termed AS25.^4^ Isothermal titration calorimetry (ITC) showed that the synthetic l-Bcr-Abl SH2 domain bound AS25 with the same affinity as the recombinantly expressed protein (Fig. 5D and Fig. S19, ESI†). Given the large interaction interface between AS25 and the Bcr-Abl SH2, the data indicate that the synthetic l-Bcr-Abl SH2 domain is correctly folded. The canonical function of SH2 domains is binding to phospho-tyrosine (pY) ligand peptides. A pY peptide that was previously shown to bind the Src SH2 domain^10^ bound the synthetic l-Bcr-Abl SH2 domain with the same affinity as the recombinantly expressed domain (Fig. 4E and Fig. S20A, ESI†). To determine functionality of the synthetic d-Bcr-Abl SH2 domain, the corresponding d-pY peptide bound the d-protein with the same affinity as the l-pY peptide to the l-Bcr-Abl SH2 domain (Fig. 4F). As expected, the d-pY peptide did not bind the recombinantly expressed l-Bcr-Abl SH2 domain (Fig. S20B, ESI†).

In summary, the *N*- and *C*-terminal fragments of the Bcr-Abl SH2 domain could be efficiently synthesized after GS-pseudoproline incorporation and suppression of MeDbz linker acetylation by exchanging acetic anhydride in the capping solution with *N*-acetoxy succinimide. The efficiently ligated and subsequently desulfurized l- and d-Bcr-Abl SH2 domain polypeptides were refolded in their native conformation and have binding properties similar to the recombinantly expressed domain.

We will use the d-Bcr-Abl SH2 domain protein for monobody development by phage- and yeast-display selection. Mirror-image monobodies will be an important step towards therapeutic application due to their higher *in vivo* stability.

## Conflicts of interest

The authors declare no competing interests.

## Supplementary Material

CB-003-D2CB00108J-s001
