# Supplementary material for: Synthesis of the l- and d-SH2 domain of the leukaemia oncogene Bcr-Abl
Source: RSC Chem Biol. 2022 Jul 6;3(8):1008–12. doi: 10.1039/d2cb00108j (PMC9347351; doi:10.1039/d2cb00108j)
Supplement: CB-003-D2CB00108J-s001 [file CB-003-D2CB00108J-s001.pdf]

Electronic Supplementary Material (ESI) for RSC Chemical Biology.
This journal is © The Royal Society of Chemistry 2022

Supporting Information

Synthesis of the L- and D-SH2 domain of the leukaemia oncogene Bcr-Abl

Nina Schmidt,[a] Frank Abendroth,[b] Olalla Vázquez[b,c *]
and Oliver Hantschel[a*]

a Institute of Physiological Chemistry, University of Marburg, 35032 Marburg, Germany.

b Faculty of Chemistry, University of Marburg, 35032 Marburg, Germany.

c Center for Synthetic Microbiology (SYNMIKRO), University of Marburg, 35032 Marburg, Germany.

* E-mail: oliver.hantschel@uni-marburg.de, olalla.vazquez@staff.uni-marburg.de

Table of Contents

1 Abbreviations, materials and general procedures S3

2 Synthesis of the Bcr-Abl SH2 domain and Fmoc-Gly-D-Ser(psiMe,Mepro)-OH S8

2.1 Crude *N*-terminal Bcr-Abl SH2 peptide (L-1a) S9

2.2 Crude *N*-terminal Bcr-Abl SH2 peptide (L-1b) S10

2.3 Crude *N*-terminal Bcr-Abl SH2 peptide (L-1c) S11

2.4 Crude *N*-terminal Bcr-Abl SH2 peptide (L-1d) S12

2.5 Activated *N*-terminal Bcr-Abl SH2 peptide (L-2) S13

2.6 Mirror-image of activated *N*-terminal Bcr-Abl SH2 peptide (D-2) S14

2.7 *C*-terminal Bcr-Abl SH2 peptide (L-3) S15

2.8 Mirror-image of *C*-terminal Bcr-Abl SH2 peptide (D-3) S16

2.9 Ligated Bcr-Abl SH2 A198C (L-4) S17

2.10 Mirror-image of ligated Bcr-Abl SH2 A198C (D-4) S18

2.11 Desulfurized Bcr-Abl SH2 (L-5) S19

2.12 Mirror-image of desulfurized Bcr-Abl SH2 (D-5) S20

2.13 Fmoc-Gly-D-Ser(psiMe,Mepro)-OH S21

3 Refolding of synthetic Bcr-Abl SH2 (L-5 and D-5) S23

4 Expression and purification of recombinant Bcr-Abl SH2 .... S24

5 Expression and purification of recombinant His_{10}-Flag-AS25 .... S25

6 Isothermal titration calorimetry (ITC) measurements with His_{10}-Flag-AS25 .... S26

7 ITC measurements with phospho peptide pYEEI S27

8 Circular dichroism (CD) measurements S28

9 Nano differential scanning fluorimetry (nanoDSF) measurements S29

10 References S30

1 Abbreviations, materials and general procedures

Abbreviations:

Ac_2O	acetic anhydride
Bcr-Abl	fusion product of breakpoint cluster region and Abelson tyrosine kinase
CD	circular dichroism
DCE	1,2-dichloroethane
DCM	dichloromethane
DIC	*N*,*N*-diisopropylcarbodiimide
DIPEA	*N*,*N*-diisopropylethylamine
DMF	*N*,*N*-dimethylformamide
EDT	1,2-ethanedithiol
EDTA	ethylenediaminetetraacetic acid
ESI-MS	electrospray ionization mass spectrometry
Et_2O	diethyl ether
FAM	5-carboxyfluoresceine
Fmoc	9-fluorenylmethoxycarbonyl
HATU	1-[bis(dimethylamino)methylene]-1H-1,2,3-triazolo[4,5-b]pyridinium 3-oxide
HBTU	3-[bis(dimethylamino)methyliumyl]-3H-benzotriazol-1-oxide
HEPES	4-(2-hydroxyethyl)-1-piperazineethanesulfonic acid
HR-MS	high-resolution mass spectrometry
IPTG	isopropyl β-D-1-thiogalactopyranoside
ITC	isothermal titration calorimetry
LB	lysogeny broth
m/z	mass-to-charge ratio
MeCN	acetonitrile
MeDbz	3-amino-4-(methylamino)-benzoic acid (Dawson linker)
MeNbz	*N*-acyl-*N′*-methylacylurea
MPAA	4-mercaptophenyl acetic acid
MRE	mean residue ellipticity
$MS\text{-}ESI^+$	electrospray ionization mass spectrometry in positive ion mode
nanoDSF	nano differential scanning fluorimetry
NCL	native chemical ligation
Ni-NTA	nickel nitrilotriacetic acid

NMM	*N*-methyl morpholine
NMP	*N*-methyl pyrrolidone
PBS	phosphate buffered saline
PyBOP	benzotriazol-1-yloxytripyrrolidinophosphonium
RP-HPLC	reverse phase high performance liquid chromatography
rpm	revolutions per minute
RT	room temperature
SEC	size exclusion chromatography
SPPS	solid phase peptide synthesis
TCEP	tris(2-carboxyethyl)phosphine
TFA	trifluoroacetic acid
TIS	triisopropylsilane
Tris	tris(hydroxymethyl)aminomethane

Materials: All commercially purchased reagents were used without further purification as delivered from the corresponding company. The respective reagents were purchased from the following companies: guanidine-HCl biochemical grade, NaCl, Na_2HPO_4 x 2 H_2O, L-glutathione, HEPES, Tris, imidazole, DIPEA, 2,6-lutidine, pyridine, NMM, DIC, piperidine and LC-MS grade TFA from Carl Roth (Germany); TentaGel S RAM resin, NMP and Fmoc-MeDbz-OH from Iris Biotech (Germany); Fmoc-protected L-amino acids, HBTU and OxymaPure from Carbolution (Germany); VA-044 x 2HCl and TCEP-HCl from TCI (Germany); 4-nitrophenyl chloroformate and TFA for peptide cleavage from Acros Organics (USA); D-biotin from J&K Scientific (Germany); Fmoc-protected D-amino acids from Merck (Germany); *N*-acetoxy succinimide from ChemPur (Germany); MeCN as HPLC LC-MS grade, DMF anhydrous and 1,2-dichloro ethane from VWR (France); Fmoc-Gly-Ser(psiMe,Mepro)-OH, MPAA, EDT, TIS, DEE, HPLC-grade DCM, kanamycin sulfate, IPTG and LB broth from Sigma-Aldrich (Germany); Gibco 10x PBS and Slide-A-Lyzer 3.5K Dialysis Cassettes G2 (#87722, #87723, #87724) from Thermo Fisher Scientific (USA); formic acid optima from Fisher Chemical (USA); EDTA-free protease inhibitor tablets and DNase I from Roche (Switzerland); HATU and PyBOP from BLDpharm (Germany); microscale columns with PTFE filter (#35.091) from CEM (USA). DMF was employed as peptide grade (Iris Biotech, Germany). The FAM-labeled L- and D-pYEEI peptides for ITC measurements were ordered from Peptide Synthetics (UK). Water was purified with a Milli-Q Ultra-Pure (TKA, Germany) or Milli-Q Advantage A10 (Merck Millipore, Germany) Water Purification System.

Solid phase peptide synthesis (SPPS): Both L- and D-peptides were prepared with the same protocol as described below by automated synthesis using the Fmoc solid phase strategy, unless noted otherwise. The synthesis was performed using microscale columns with PTFE filter (CEM) in a ResPep SLi (Intavis) parallel synthesizer. TentaGel S RAM resin (0.22 mmol/g) was used for synthesis.

<u>Protocol A:</u> This protocol was used to synthesize the *N*-terminal Bcr-Abl SH2 peptides. The reagent amounts correspond to 5 µmol scale synthesis.

Manual coupling of Fmoc-MeDbz-OH: The corresponding amount of resin for a 5 µmol scale synthesis (~23 mg) was weighted in microscale columns and 200 µL DMF were added. The resin was swelled for 30 min. DMF/piperidine (4:1, 200 µL) was added to the resin. After 5 min, the procedure was repeated once. The resin was washed (6 × 200 µL DMF) and 200 µL of a 0.25 M solution of Fmoc-MeDbz-OH

(10 eq.), 0.25 M HBTU (10 eq.) and 0.5 M DIPEA (20 eq.) was added to the resin. The procedure was repeated after 30 min and the resin was washed (6 × 200 µL DMF).

Automated washing of the resin: The resin was washed (4 × 300 µL DMF) prior to synthesis start by the device.

Automated deprotection of the Fmoc group: DMF/piperidine (4:1, 150 µL) was added to the resin. After 5 min, the procedure was repeated once. The resin was washed (1 × 300 µL DMF, 3 × 225 µL DMF).

Automated coupling of amino acid monomers: Standard amino acids were coupled by charging the reactor with 53 µL of a solution of the corresponding Fmoc-amino acid (0.5 M solution in NMP, 5 eq.), 15 µL OxymaPure (6 eq.), 13 µL DIC (5 eq.) and 29 µL NMP. The resulting solution was incubated for 20 min at 50 °C, the resin was filtered and subjected to a recoupling step following the same procedure. Fmoc-Gly-(L- or D-)-Ser(psiMe,Mepro)-OH was coupled by charging the reactor with a solution of the corresponding Fmoc-pseudoproline (0.5 M solution in NMP, 5 eq.), HATU (4.8 eq.) and NMM (13 eq.). The resulting solution was incubated for 30 min, the resin was filtered and subjected to a recoupling step following the same procedure.

Capping Protocol A1: Ac_2O/2,6-lutidine/DMF (5:15:80, 120 µL) was added for 8 min. Then, the resin was washed (3 × 225 µL DMF). The capping reagent was exchanged for a fresh-prepared solution every 48 h. These conditions were used for peptides without the MeDbz linker and before capping with *N*-acetoxy succinimide was implemented.

Capping Protocol A2: 0.5 M *N*-acetoxy succinimide in pyridine/DMF (1:6, 120 µL) was added for 8 min. Then, the resin was washed (3 × 225 µL DMF). The capping reagent was exchanged for a fresh-prepared solution every 48 h. These conditions were used for peptides with the MeDbz linker.

Manual coupling of D-biotin to N-terminal L- and D-Bcr-Abl SH2 peptide after completion of synthesis: 500 µL of a 0.1 M solution of D-biotin (10 eq.), 0.1 M PyBOP (10 eq.) and 0.2 M NMM (20 eq.) were added to the resin. After 30 min, the procedure was repeated once with 500 µL of a 0.1 M solution of D-biotin (10 eq.), 0.1 M HATU (10 eq.) and 0.2 M NMM (20 eq.). Then, the resin was washed (6 × 200 µL DMF, 6 × 200 µL DCM).

Automated final Fmoc deprotection and washing of resin: DMF/piperidine (4:1, 150 µL) was added to the resin. After 5 min, the procedure was repeated twice. The resin was finally washed (4 × 300 µL DMF, 4 × 150 µL ethanol, 5 x 150 µL DCM).

<u>Protocol B:</u> This protocol was used to synthesize the *C*-terminal Bcr-Abl SH2 peptides. The reagent amounts correspond to 5 µmol scale synthesis.

Automated washing of the resin: The corresponding amount of resin for a 5 µmol scale synthesis (~23 mg) was weighted in microscale columns. Then, the resin was washed (4 × 300 µL DMF) prior to synthesis start by the device.

Automated deprotection of the Fmoc group: DMF/piperidine (4:1, 150 µL) was added to the resin. After 5 min, the procedure was repeated once. The resin was washed (1 × 300 µL DMF, 3 × 225 µL DMF).

Automated coupling of amino acid monomers: Standard amino acids were coupled by charging the reactor with 53 µL of a solution of the corresponding Fmoc-amino acid (0.5 M solution in NMP, 5 eq.), 15 µL OxymaPure (6 eq.), 13 µL DIC (5 eq.) and 29 µL NMP. The resulting solution was incubated for 20 min at 50 °C, the resin was filtered and subjected to a recoupling step following the same procedure.

Capping Protocol A1: Ac_2O/2,6-lutidine/DMF (5:15:80, 120 µL) was added for 8 min. Then, the resin was washed (3 × 225 µL DMF). The capping reagent was exchanged for a fresh-prepared solution every 48 h. These conditions were used for peptides without the MeDbz linker.

Automated final Fmoc deprotection and washing of resin: DMF/piperidine (4:1, 150 µL) was added to the resin. After 5 min, the procedure was repeated twice. The resin was finally washed (4 × 300 µL DMF, 4 × 150 µL ethanol, 5 x 150 µL DCM).

Final cleavage for both protocols (A and B): The resin was treated with the cleavage cocktail (92.5% TFA, 2.5% H_2O, 2.5% TIS, 2.5% EDT up to 2 mL of cocktail for ~30 mg of resin). The resulting suspension was shaken for 2.5 h and the resin was filtered off. The resulting filtrated solution was concentrated with nitrogen gas until less than 1 mL of cleavage cocktail was left, and added to ice-cold Et_2O (10 mL of Et_2O for each 1 mL of TFA). After 10 min, the mixture was centrifuged (8000 rpm, 10 min, 4 °C), decantated, and the solid was again suspended in 10 mL of fresh ice-cold Et_2O, sonicated and centrifuged again. This procedure was repeated once and the solid residue was dried. Then, the *N*-terminal Bcr-Abl SH2 peptide was dissolved in H_2O/MeCN (50:50, v/v) with addition of 0.1% TFA, whereas the *C*-terminal Bcr-Abl SH2 peptide was dissolved in H_2O/MeCN (85:15, v/v) with addition of 0.1% TFA. The peptides were purified by semipreparative reverse-phase (RP)-HPLC.

Purification: The peptides were purified by semipreparative HPLC, performed on a Thermo Scientific Dionex UltiMate 3000 series instrument (column: *Kinetex EVO C18* 5 µm, 150 × 21.2 mm, pore size 100 Å, flow rate of 20 mL/min) using eluents A (99.9% H_2O, 0.1% TFA) and B (99.9% acetonitrile, 0.1% TFA) in the corresponding linear gradient as written for each peptide (sections 2.5 – 2.12). Detection of the signals was achieved with a UV-detector at wavelength 220 nm. The collected fractions were lyophilized and stored at -20 °C.

Characterization of probes: Analytical HPLC chromatograms were performed on three different systems:

a) An Agilent 1260 infinity II LC system equipped with a 6120B Single Quadrupole mass spectrometer (column: *ZORBAX Eclipse XDB-C18* 5 µm, 150 × 4.6 mm, pore size 80 Å). A flow rate of 1 mL/min and a column temperature of 55 °C were used. Detection of signals were achieved with a UV detector at wavelength 220 nm. Electrospray Ionization Mass Spectrometry (ESI-MS) was performed by direct injection on the 6120B Single Quadrupole mass spectrometer in positive scan mode. The following method was used:

Tab. S1: HPLC method used on the Agilent LC system (a). Measurements of analytical data with this method are mentioned in the footnote of the corresponding chromatograms.

HPLC Method	Eluent A	Eluent B	Gradient
A	99.95% H_2O, 0.05% TFA	99.97% acetonitrile, 0.03% TFA	5 – 75% B in 25 min

b) The UHPLC system was the Thermo Scientific Dionex UltiMate 3000 RS instrument (column: *Kinetex EVO C18* 1.7 µm, 50 × 2.1 mm, pore size 100 Å). A flow rate of 0.5 mL/min and a column temperature of 40 °C were used. Detection of signals was achieved with a UV detector at wavelength 220 nm. ESI-MS was performed by direct injection on a Thermo Scientific MSQ Plus mass spectrometer in positive scan mode. The following methods were used:

Tab. S2: HPLC method used on the Thermo Scientific LC system (b). Measurements of analytical data with these methods are mentioned in the footnote of the corresponding chromatograms.

HPLC Method	Eluent A	Eluent B	Gradient
B	99.95% H_2O, 0.05% TFA	99.97% acetonitrile, 0.03% TFA	5 – 75% B in 25 min
C	99.9% H_2O, 0.1% formic acid	99.9% acetonitrile, 0.1% formic acid	5 – 75% B in 25 min
D	99.95% H_2O, 0.05% TFA	99.97% acetonitrile, 0.03% TFA	5 – 30% B in 25 min

c) High-resolution electrospray ionization mass spectra (HR-MS) were acquired at the Mass Spectrometry Facility of the Philipps-University Marburg with an LTQ-FT Ultra mass spectrometer (Thermo Fischer Scientific). The resolution was set to 100000.

2 Synthesis of the Bcr-Abl SH2 domain and Fmoc-Gly-D-Ser(psiMe,Mepro)-OH

The two peptide fragments for the synthesis of the Bcr-Abl SH2 domain were designed as depicted in Fig. S1. An overview of the synthesized sequences is listed in Tab. S1.

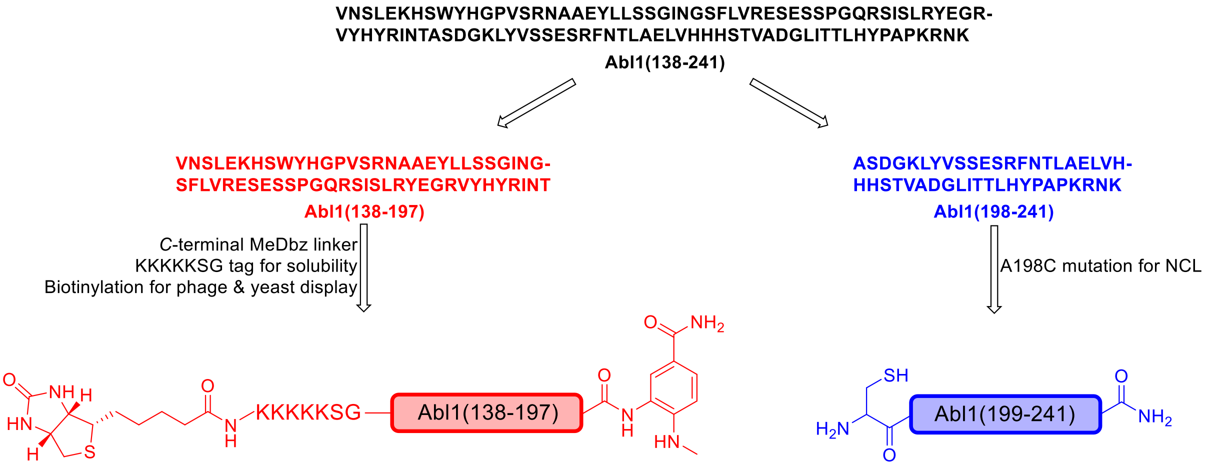

Fig. S1: Depiction of the two *N*- and *C*-terminal Bcr-Abl SH2 domain fragments based on the sequence of Bcr-Abl(138-241).

Tab. S3: Sequences of the synthesized peptide fragments, which were ligated and subsequently desulfurized to yield biotinylated Bcr-Abl(138-241). The underlined residues indicate the incorporation of the Fmoc-Gly-Ser(psiMe,Mepro)-OH pseudoproline.

Peptide	Sequence	Mass [Da]
**1a**	Biotin-KKKKKSG-VNSLEKHSWYHGPVSRNAAEYLLSSGINGSFLV-RESESSPGQRSISLRYEGRVYHYRINT-CONH$_2$	7866.8
**1b**	Biotin-KKKKKSG-VNSLEKHSWYHGPVSRNAAEYLLSSGINGSFLV-RESESSPGQRSISLRYEGRVYHYRINT-CONH$_2$	7866.8
**1c**	Biotin-KKKKKSG-VNSLEKHSWYHGPVSRNAAEYLLSSGINGSFLV-RESESSPGQRSISLRYEGRVYHYRINT-MeDbz-CONH$_2$	8015.1
**1d**	Biotin-KKKKKSG-VNSLEKHSWYHGPVSRNAAEYLLSSGINGSFLV-RESESSPGQRSISLRYEGRVYHYRINT-MeDbz-CONH$_2$	8015.1
**2**	Biotin-KKKKKSG-VNSLEKHSWYHGPVSRNAAEYLLSSGINGSFLV-RESESSPGQRSISLRYEGRVYHYRINT-MeNbz-CONH$_2$	8041.0
**3**	H$_2$N-CSDGKLYVSSESRFNTLAELVHHHSTVADGLITTLHYPAPKR-NK-CONH$_2$	4893.5
**4**	Biotin-KKKKKSG-VNSLEKHSWYHGPVSRNAAEYLLSSGINGSFLV-RESESSPGQRSISLRYEGRVYHYRINTCSDGKLYVSSESRFNTLAEL-VHHHSTVADGLITTLHYPAPKRNK-CONH$_2$	12743.4
**5**	Biotin-KKKKKSG-VNSLEKHSWYHGPVSRNAAEYLLSSGINGSFLV-RESESSPGQRSISLRYEGRVYHYRINTASDGKLYVSSESRFNTLAEL-VHHHSTVADGLITTLHYPAPKRNK-CONH$_2$	12711.3

2.1 Crude *N*-terminal Bcr-Abl SH2 peptide (L-1a)

Peptide **L-1a** was synthesized according to SPPS Protocol A without the usage of the L-pseudoproline Fmoc-Gly-L-Ser(psiMe,Mepro)-OH and the Capping Protocol A1. Truncations occurred between I_{164} and N_{165} (Fig. S2 B, marked with *) as well as N_{165} and G_{166} (Fig. S2 B, marked with **).

HRMS-ESI$^+$ (m/z): $[M+8H]^{8+}$ calcd.: 984.2651; found: 984.2684.

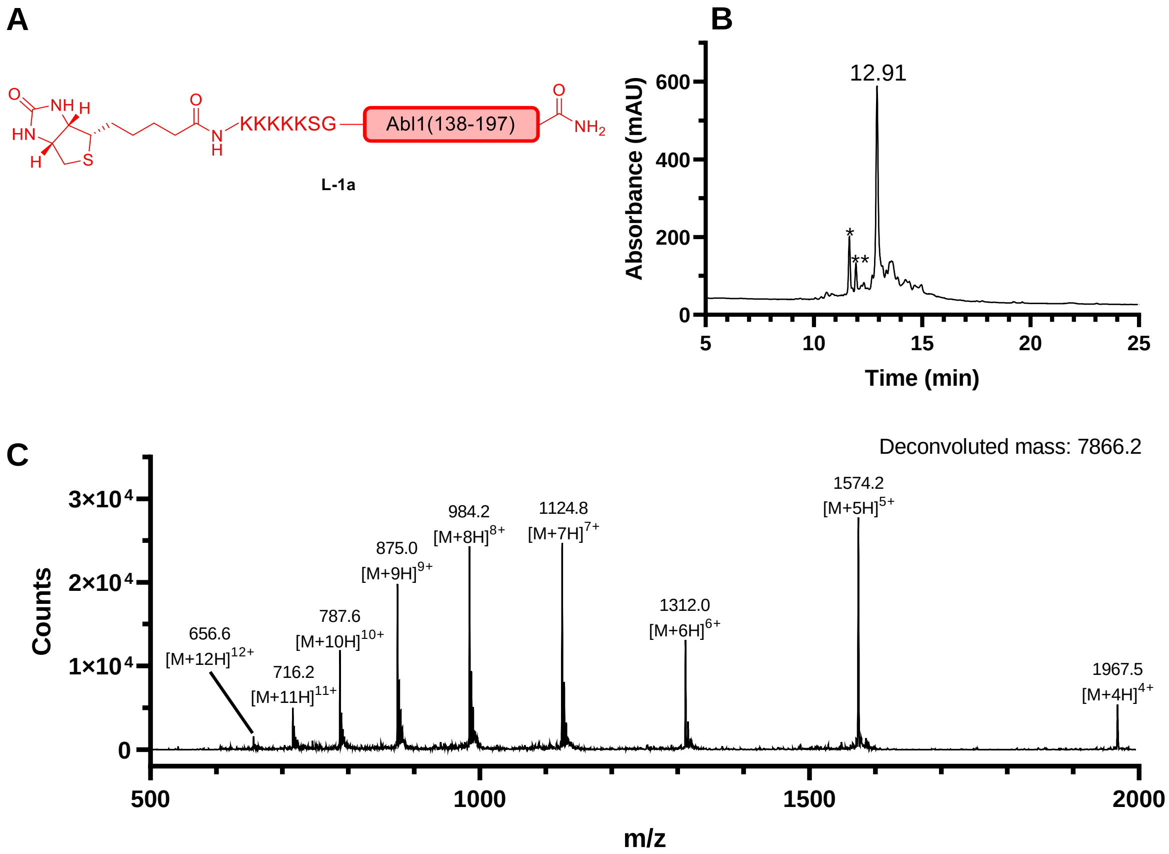

Fig. S2: A) Structure of peptide **L-1a**; **B)** Analytical RP-HPLC chromatogram of peptide crude (HPLC method A, Tab. S1), * corresponds to the truncation between I_{164} and N_{165}, ** corresponds to the truncation between N_{165} and G_{166}; **C)** MS-ESI$^+$ spectrum of **L-1a** (HPLC Method A, Tab. S1), mass spectra were manually deconvoluted with ESIprot Online.[1]

2.2 Crude *N*-terminal Bcr-Abl SH2 peptide (L-1b)

Peptide **L-1b** was synthesized according to SPPS Protocol A with the usage of the L-pseudoproline Fmoc-Gly-L-Ser(psiMe,Mepro)-OH and the Capping Protocol A1.

HRMS-ESI$^+$ (m/z): $[M+8H]^{8+}$ calcd.: 984.2651; found: 984.2696.

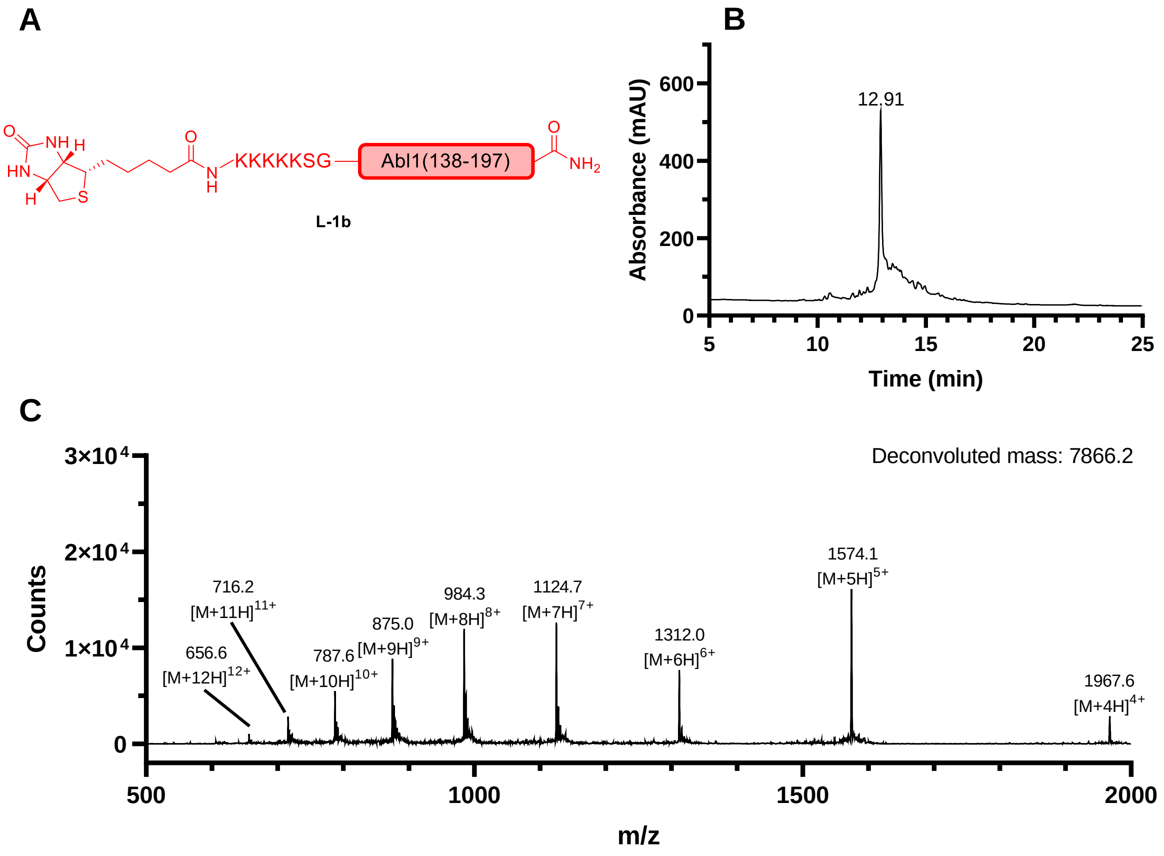

Fig. S3: A) Structure of peptide **L-1b**; **B)** Analytical RP-HPLC chromatogram of peptide crude (HPLC method A, Tab. S1); **C)** MS-ESI$^+$ spectrum of **L-1b** (HPLC Method A, Tab. S1), mass spectra were manually deconvoluted with ESIprot Online.[1]

2.3 Crude *N*-terminal Bcr-Abl SH2 peptide (L-1c)

Peptide **L-1c** was synthesized according to SPPS Protocol A with the usage of the L-pseudoproline Fmoc-Gly-L-Ser(psiMe,Mepro)-OH and the Capping Protocol A1. The usage of this capping protocol led to acetylation of the MeDbz linker as seen in the MS-ESI$^+$ spectrum.

HRMS-ESI$^+$ (m/z): $[M+11H]^{11+}$ calcd.: 729.5643; found: 729.5708.

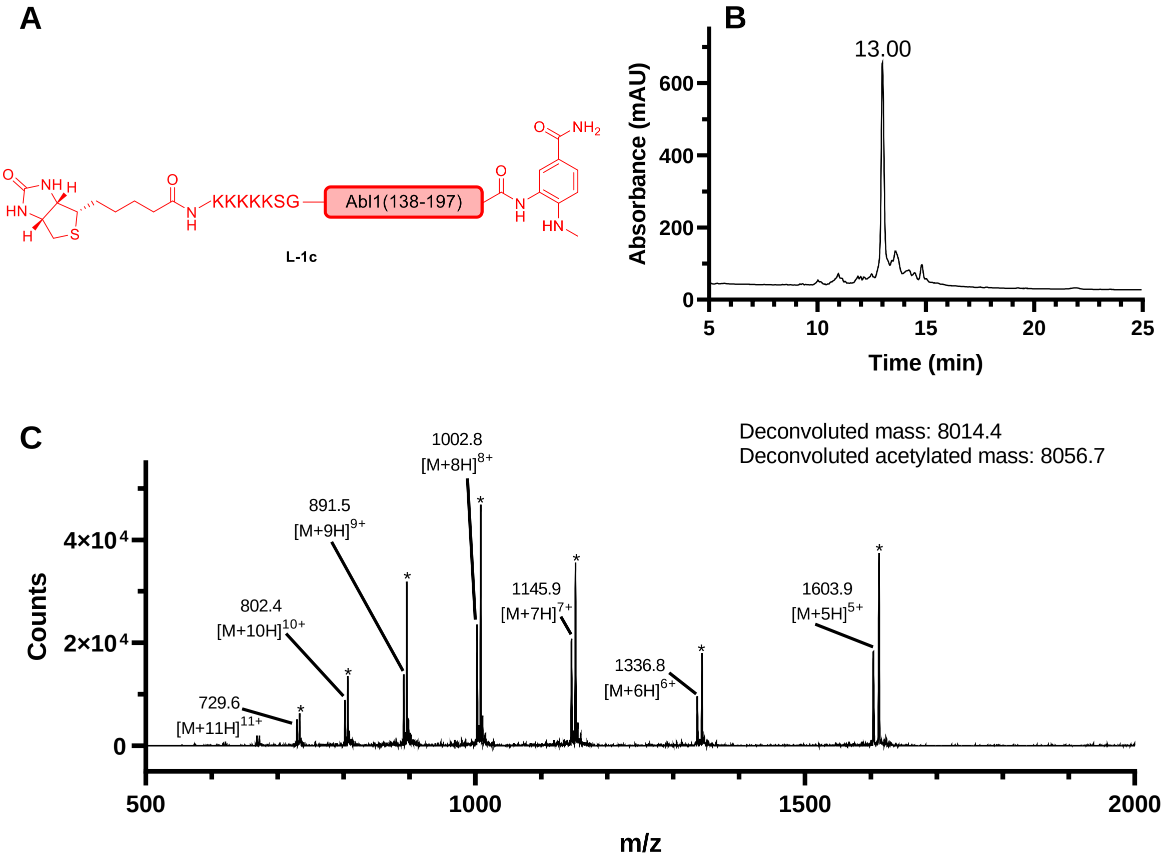

Fig. S4: A) Structure of peptide **L-1c**; **B)** Analytical RP-HPLC chromatogram of peptide crude (HPLC method A, Tab. S1); **C)** MS-ESI$^+$ spectrum of **L-1c** (HPLC Method A, Tab. S1), peaks marked with * correspond to the peptide with acetylated MeDbz linker (733.5 $[M+11H]^{11+}$, 806.8 $[M+10H]^{10+}$, 896.1 $[M+9H]^{9+}$, 1008.1 $[M+8H]^{8+}$, 1151.9 $[M+7H]^{7+}$, 1343.7 $[M+6H]^{6+}$, 1612.3 $[M+5H]^{5+}$), mass spectra were manually deconvoluted with ESIprot Online.[1]

2.4 Crude *N*-terminal Bcr-Abl SH2 peptide (L-1d)

Peptide **L-1d** was synthesized according to SPPS Protocol A with the usage of the L-pseudoproline Fmoc-Gly-L-Ser(psiMe,Mepro)-OH and the capping protocol A2. The usage of this capping protocol suppressed the acetylation of the MeDbz linker as observable in the MS-ESI$^+$ spectrum.

HRMS-ESI$^+$ (m/z): $[M+11H]^{11+}$ calcd.: 729.5642; found: 729.5674.

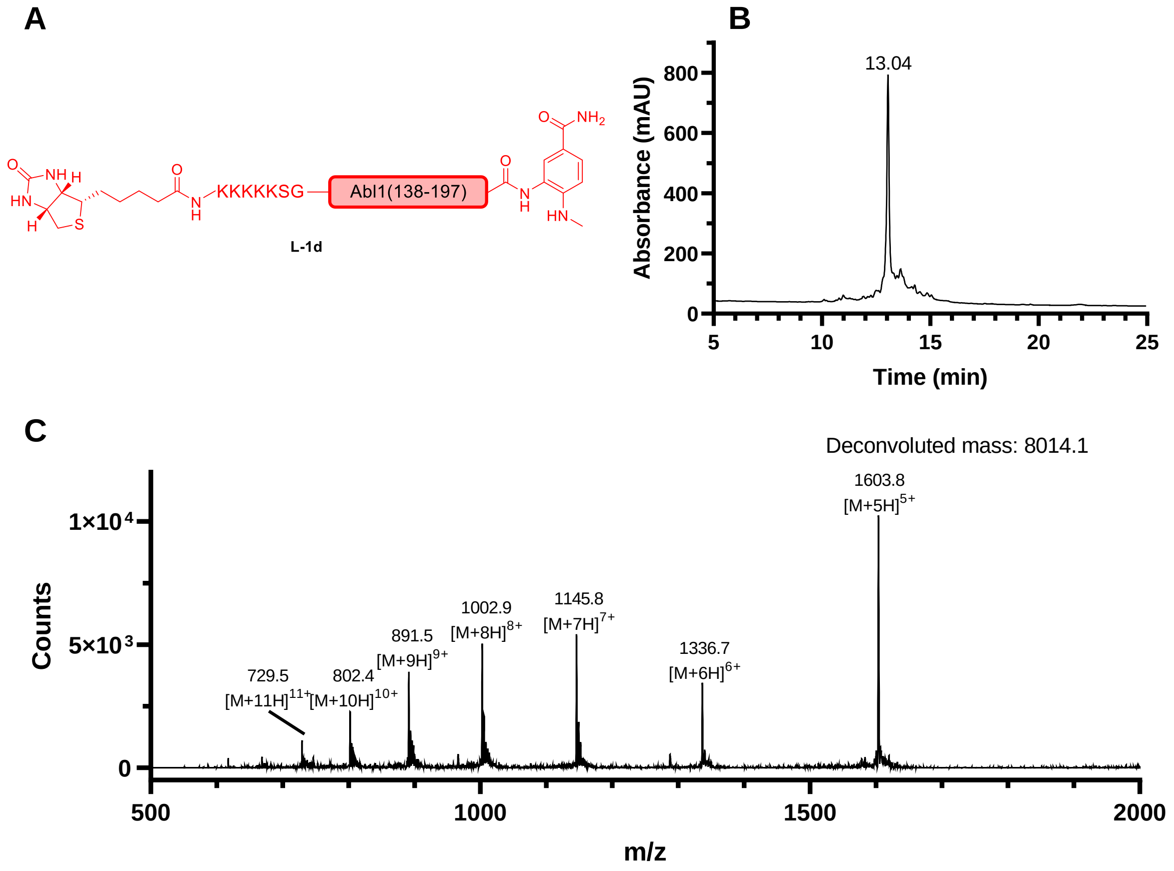

Fig. S5: A) Structure of peptide **L-1d**, **B)** Analytical RP-HPLC chromatogram of peptide crude (HPLC method A, Tab. S1), **C)** MS-ESI$^+$ spectrum of **L-1d** (HPLC Method A, Tab. S1), mass spectra were manually deconvoluted with ESIprot Online.[1]

2.5 Activated *N*-terminal Bcr-Abl SH2 peptide (L-2)

Peptide **L-2** was synthesized according to SPPS Protocol A with the usage of the L-pseudoproline Fmoc-Gly-L-Ser(psiMe,Mepro)-OH and the Capping Protocol A2. The MeDbz linker activation was performed on resin, which was treated with 4-nitrophenyl chloroformate (2 eq., 20 mM) dissolved in 1500 µL 1,2-dichloroethane (DCE) for 45 min. The reaction was repeated twice. After washing the resin (6 × DCE, 6 × anhydrous DMF), 1500 µL of DIPEA (50 eq., 0.5 M) in anhydrous DMF were added. The reaction proceeded for 15 min, repeated for 30 min and then conducted a third time for 15 min. After cleavage of the peptide from the resin, the precipitated peptide was dissolved in H_2O/MeCN (50:50, v/v) with addition of 0.1% TFA and purified via semipreparative RP-HPLC using a linear gradient of 5-40% MeCN with 0.1% TFA over 30 min. After lyophilization, 13.9 mg (1.42 µmol, 10%, 15 µmol scale) of the desired peptide were obtained as TFA salt.

HRMS-ESI$^+$ (m/z): $[M+8H]^{8+}$ calcd.: 1006.0205; found: 1006.0234.

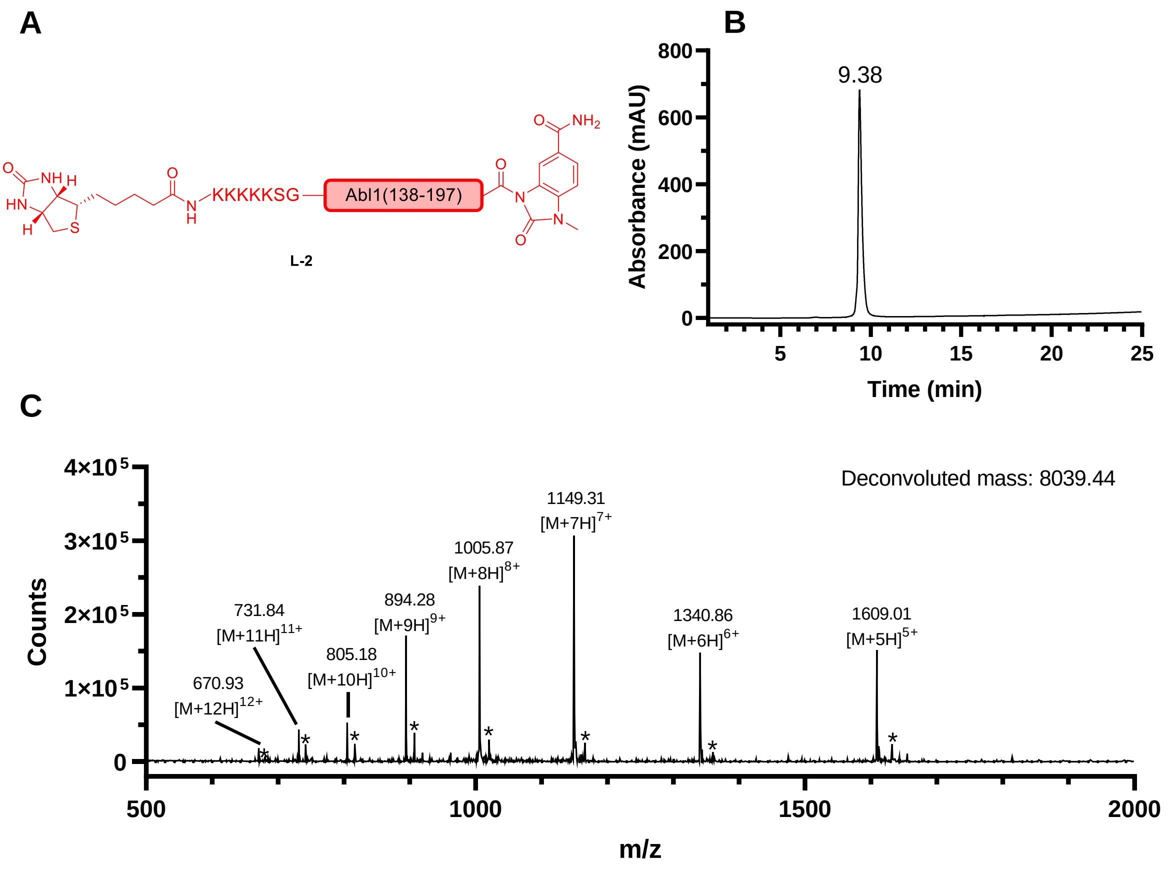

Fig. S6: A) Structure of peptide **L-2**; **B)** Analytical RP-HPLC chromatogram of purified peptide (HPLC method B, Tab. S2); **C)** MS-ESI$^+$ spectrum of **L-2** (HPLC Method B, Tab. S2), peaks marked with * correspond to the peptide with TFA (680.36 $[M+12H]^{12+}$, 742.13 $[M+11H]^{11+}$, 816.58 $[M+10H]^{10+}$, 907.14 $[M+9H]^{9+}$, 1020.33 $[M+8H]^{8+}$, 1166.21 $[M+7H]^{7+}$, 1359.98 $[M+6H]^{6+}$, 1632.12 $[M+5H]^{5+}$), mass spectra were manually deconvoluted with ESIprot Online.[1]

2.6 Mirror-image of activated *N*-terminal Bcr-Abl SH2 peptide (D-2)

Peptide **D-2** was synthesized according to SPPS Protocol A with the usage of the D-pseudoproline Fmoc-Gly-D-Ser(psiMe,Mepro)-OH and the Capping Protocol A2. The MeDbz linker activation was performed on resin, which was treated with 4-nitrophenyl chloroformate (2 eq., 20 mM) dissolved in 1500 µL of DCE for 45 min. The reaction was repeated twice. After washing the resin (6 × DCE, 6 × anhydrous DMF), 1500 µL of DIPEA (50 eq., 0.5 M) in anhydrous DMF were added. The reaction proceeded for 15 min, was repeated for 30 min and then conducted a third time for 15 min. After cleavage of the peptide from the resin, the precipitated peptide was dissolved in H_2O/MeCN (50:50, v/v) with addition of 0.1% TFA and purified via semipreparative RP-HPLC using a linear gradient of 5-40% MeCN with 0.1% TFA over 30 min. After lyophilization, 16.9 mg (1.73 µmol, 12%, 15 µmol scale) of the desired peptide were obtained as TFA salt.

HRMS-ESI$^+$ (m/z): $[M+10H]^{10+}$ calcd.: 805.0179; found: 805.0206.

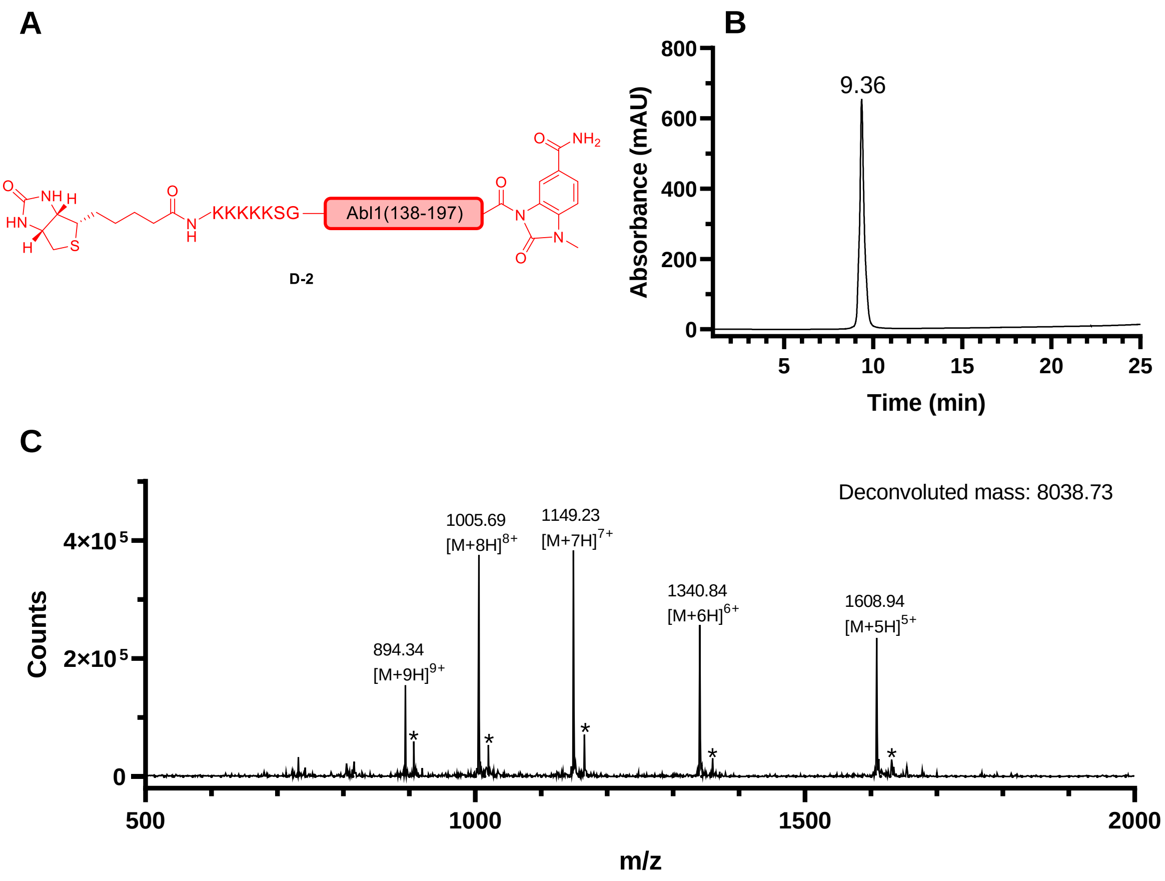

Fig. S7: A) Structure of peptide **D-2**; **B)** Analytical RP-HPLC chromatogram of purified peptide (HPLC method B, Tab. S2); **C)** MS-ESI$^+$ spectrum of **D-2** (HPLC Method B, Tab. S2), peaks marked with * correspond to the peptide with TFA (906.99 $[M+9H]^{9+}$, 1020.17 $[M+8H]^{8+}$, 1165.71 $[M+7H]^{7+}$, 1360.25 $[M+6H]^{6+}$, 1631.66 $[M+5H]^{5+}$), mass spectra were manually deconvoluted with ESIprot Online.[1]

2.7 *C*-terminal Bcr-Abl SH2 peptide (L-3)

Peptide **L-3** was synthesized according to SPPS Protocol B and Capping Protocol A1. After cleavage of the peptide from the resin, the precipitated peptide was dissolved in H_2O/MeCN (85:15, v/v) with addition of 0.1% TFA and purified via semipreparative RP-HPLC using a linear gradient of 5-40% MeCN with 0.1% TFA over 30 min. After lyophilization, 18.2 mg (3.02 µmol, 12%, 25 µmol scale) of the desired peptide were obtained as TFA salt.

HRMS-ESI$^+$ (m/z): $[M+6H]^{6+}$ calcd.: 816.4247; found: 816.4347.

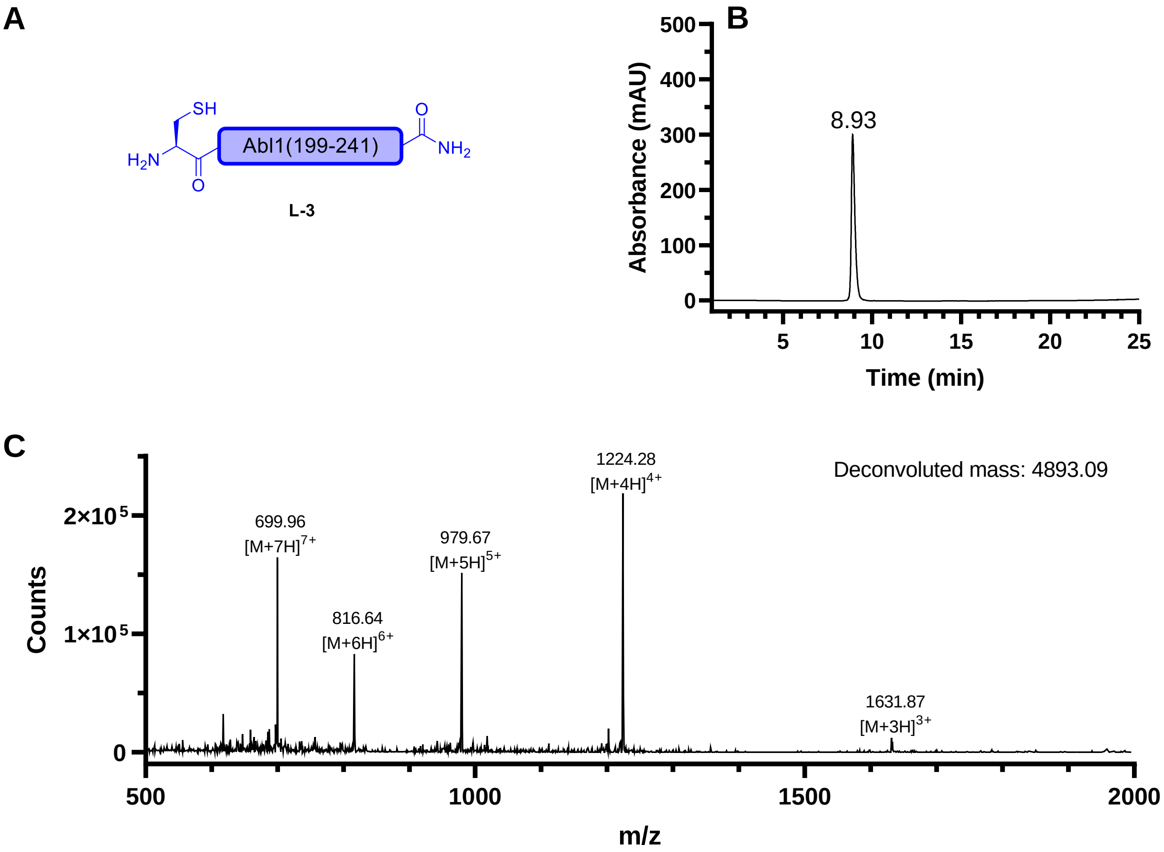

Fig. S8: A) Structure of peptide **L-3**; **B)** Analytical RP-HPLC chromatogram of purified peptide (HPLC method B, Tab. S2); **C)** MS-ESI$^+$ spectrum of **L-3** (HPLC Method B, Tab. S2), mass spectra were manually deconvoluted with ESIprot Online.[1]

2.8 Mirror-image of *C*-terminal Bcr-Abl SH2 peptide (D-3)

Peptide **D-3** was synthesized according to SPPS Protocol B and Capping Protocol A1. After cleavage of the peptide from the resin, the precipitated peptide was dissolved in H_2O/MeCN (85:15, v/v) with addition of 0.1% TFA and purified via semipreparative RP-HPLC using a linear gradient of 5-40% MeCN with 0.1% TFA over 30 min. After lyophilization, 12.4 mg (2.06 µmol, 14%, 15 µmol scale) of the desired peptide were obtained as TFA salt.

HRMS-ESI$^+$ (m/z): $[M+7H]^{7+}$ calcd.: 699.9365; found: 699.9389.

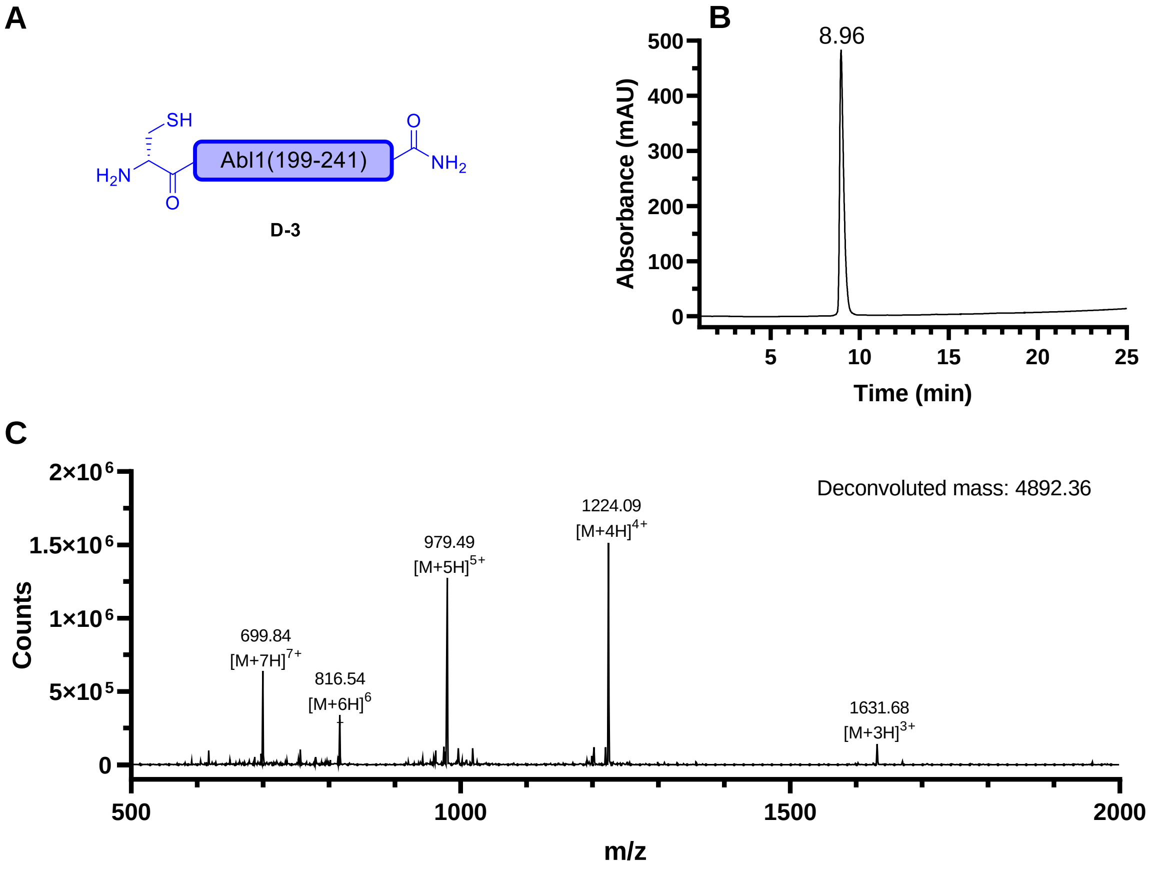

Fig. S9: A) Structure of peptide **D-3**; **B)** Analytical RP-HPLC chromatogram of purified peptide (HPLC method B, Tab. S2); **C)** MS-ESI$^+$ spectrum of **D-3** (HPLC Method B, Tab. S2), mass spectra were manually deconvoluted with ESIprot Online.[1]

2.9 Ligated Bcr-Abl SH2 A198C (L-4)

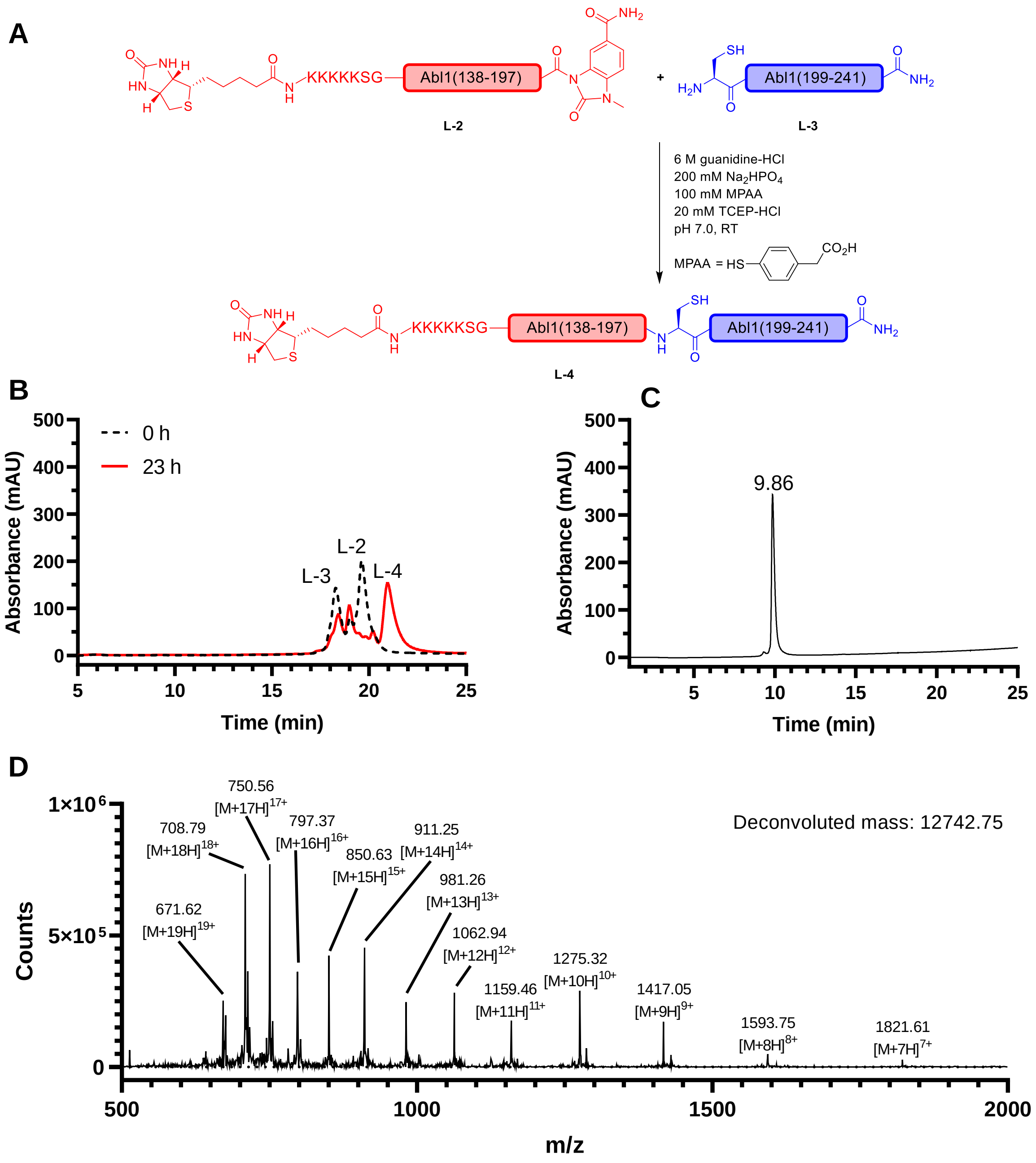

Fig. S10: A) Reaction scheme of the native chemical ligation (NCL) between peptides **L-2** and **L-3** to obtain **L-4**; **B)** Analytical RP-HPLC chromatogram of NCL crude (HPLC Method D, Tab. S2); **C)** Analytical RP-HPLC chromatogram of purified ligation product (HPLC Method B, Tab. S2); **D)** MS-ESI$^+$ spectrum of **L-4** (same sample as in **C** was remeasured with HPLC Method C, Tab. S2), mass spectra were manually deconvoluted with ESIprot Online.[1]

Peptides **L-2** (1 eq.) and **L-3** (1.2 eq.) were dissolved in 710 µL of freshly prepared buffer containing 6 M guanidine-HCl, 200 mM Na_2HPO_4, 100 mM MPAA and 20 mM TCEP-HCl. The pH was adjusted to 7.0 using 10 M NaOH and the ligation reaction was carried out at room temperature (RT) for 24 h. Then, a spatula tip of TCEP-HCl powder was added to the mixture. After 30 min, the crude product was purified via semipreparative RP-HPLC using a linear gradient of 5-35% MeCN containing 0.1% TFA over 30 min. After lyophilization, 11.2 mg (0.72 µmol, 51%) of the desired peptide were obtained as TFA salt.

HRMS-ESI$^+$ (m/z): $[M+15H]^{15+}$ calcd.: 850.5103; found: 850.5148.

2.10 Mirror-image of ligated Bcr-Abl SH2 A198C (D-4)

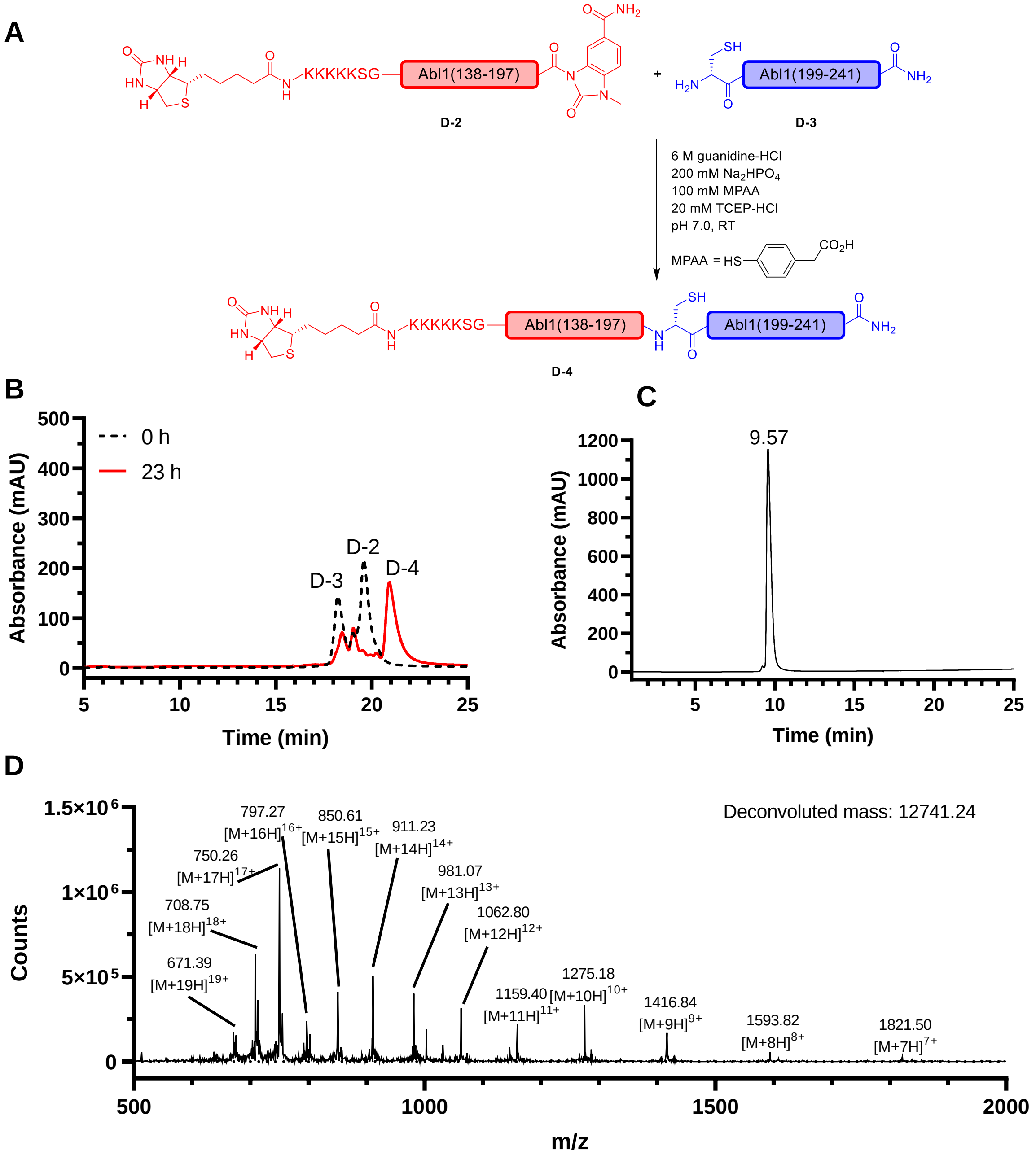

Fig. S11: A) Reaction scheme of the NCL between peptides **D-2** and **D-3** to obtain **D-4**; **B)** Analytical RP-HPLC chromatogram of NCL crude (HPLC Method D, Tab. S2); **C)** Analytical RP-HPLC chromatogram of purified ligation product (HPLC Method B, Tab. S2); **D)** MS-ESI$^+$ spectrum of **D-4** (same sample as in **C** was remeasured with HPLC Method C, Tab. S2), mass spectra were manually deconvoluted with ESIprot Online.[1]

Peptides **D-2** (1 eq.) and **D-3** (1.2 eq.) were dissolved in 864 µL of freshly prepared buffer containing 6 M guanidine-HCl, 200 mM Na_2HPO_4, 100 mM MPAA and 20 mM TCEP-HCl. The pH was adjusted to 7.0 using 10 M NaOH and the ligation reaction was carried out at RT for 24 h. Then, a spatula tip of TCEP-HCl powder was added to the mixture. After 30 min, the crude product was purified via semipreparative RP-HPLC using a linear gradient of 5-35% MeCN containing 0.1% TFA over 30 min. After lyophilization, 15.3 mg (0.99 µmol, 57%) of the desired peptide were obtained as TFA salt.

HRMS-ESI$^+$ (m/z): $[M+11H]^{11+}$ calcd.: 1159.4202; found: 1159.4234.

2.11 Desulfurized Bcr-Abl SH2 (L-5)

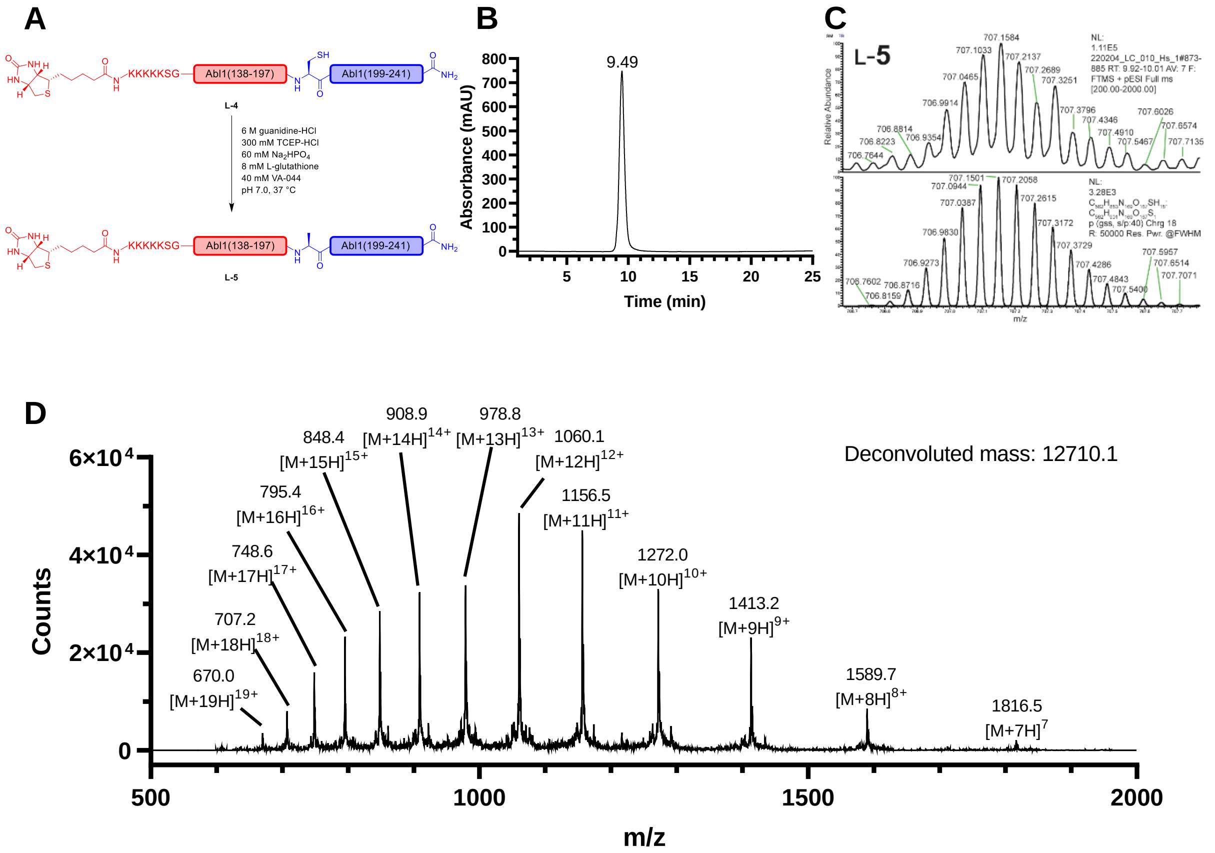

Fig. S12: A) Desulfurization reaction of **L-4** to obtain **L-5**; **B)** Analytical RP-HPLC chromatogram of purified desulfurized product (HPLC Method B; Tab. S2); **C)** High-resolution mass spectrum of **L-5**; **D)** MS-ESI$^+$ spectrum of **L-5** (same sample as in **B** was remeasured with HPLC Method A; Tab. S2), mass spectra were manually deconvoluted with ESIprot Online.[1]

Peptide **L-4** (1.5 mM) was dissolved in 480 µL of freshly prepared reaction buffer containing 6 M guanidine-HCl, 300 mM TCEP-HCl, 60 mM Na_2HPO_4, 8 mM red. L-glutathione and 40 mM VA-044. In detail, 500 mM TCEP-HCl was added to 288 µL of a 6 M guanidine-HCl/ 200 mM Na_2HPO_4 (pH 8.5) stock solution. This stock solution was mixed with 96 µL of a 6 M guanidine-HCl/ 40 mM L-glutathione stock solution and 96 µL of a 6 M guanidine-HCl/ 200 mM VA-044 stock solution to obtain 480 µL of the reaction buffer. The stock solutions can be stored at -80 °C. The pH was adjusted to 7.0 using 10 M NaOH and the desulfurization reaction was carried out at 37 °C for 16 h.[2] The crude product was purified via semipreparative RP-HPLC using a linear gradient of 5-40% MeCN containing 0.1% TFA over 15 min. After lyophilization, 8.0 mg (0.52 µmol, 72%) of the desired peptide were obtained as TFA salt.

HRMS-ESI$^+$ (m/z): $[M+18H]^{18+}$ calcd.: 707.1501; found: 707.1584.

2.12 Mirror-image of desulfurized Bcr-Abl SH2 (D-5)

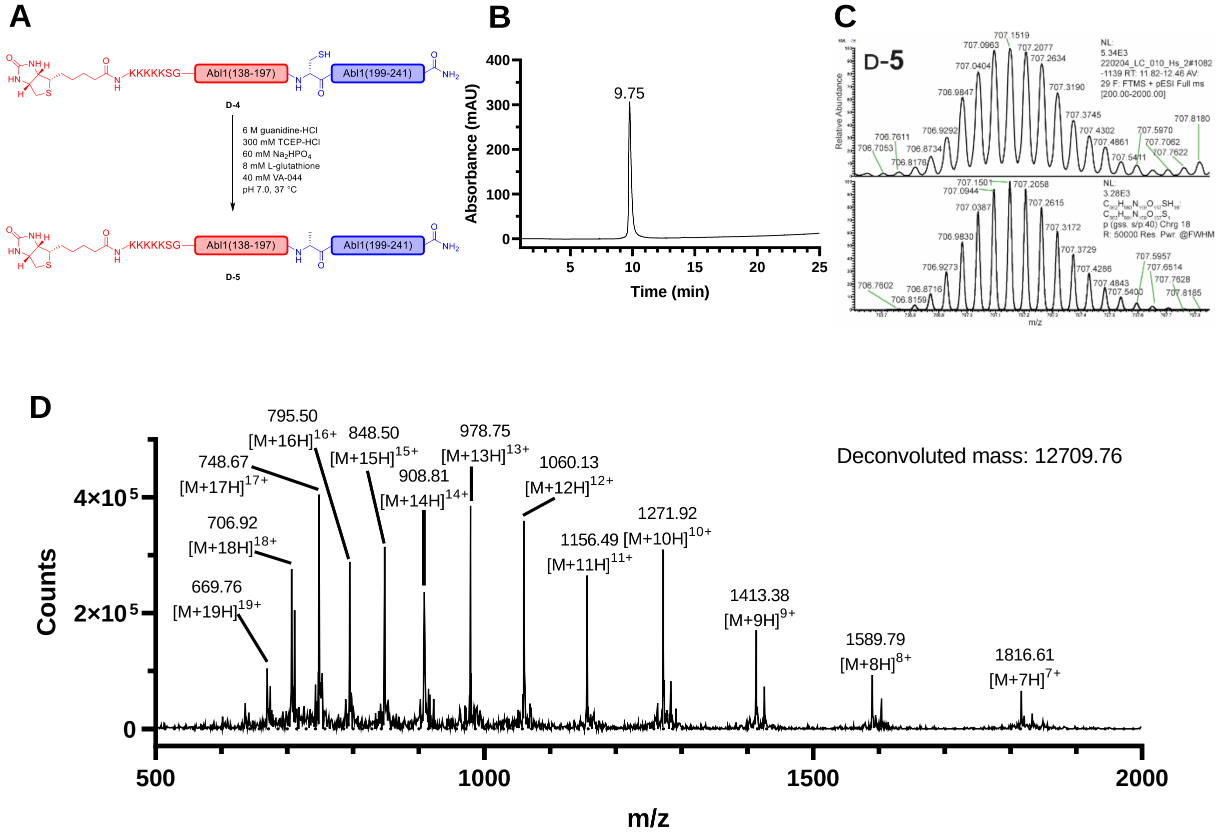

Fig. S13: A) Desulfurization reaction of **D-4** to obtain **D-5**; **B)** Analytical RP-HPLC chromatogram of purified desulfurized product (HPLC Method B; Tab. S2); **C)** High-resolution mass spectrum of **D-5**; **D)** MS-ESI$^+$ spectrum of **D-5** (same sample as in **B** was remeasured with HPLC Method C; Tab. S2). mass spectra were manually deconvoluted with ESIprot Online.[1]

Peptide **D-4** (1.5 mM) was dissolved in 655 µL of freshly prepared reaction buffer containing 6 M guanidine-HCl, 300 mM TCEP-HCl, 60 mM Na_2HPO_4, 8 mM red. L-glutathione and 40 mM VA-044. In detail, 500 mM TCEP-HCl was added to 393 µL of a 6 M guanidine-HCl/ 200 mM Na_2HPO_4 (pH 8.5) stock solution. This stock solution was mixed with 131 µL of a 6 M guanidine-HCl/ 40 mM L-glutathione stock solution and 131 µL of a 6 M guanidine-HCl/ 200 mM VA-044 stock solution to obtain 655 µL of the reaction buffer. The stock solutions can be stored at -80 °C. The pH was adjusted to 7.0 using 10 M NaOH and the desulfurization reaction was carried out at 37 °C for 16 h.[2] The crude product was purified via semipreparative RP-HPLC using a linear gradient of 5-40% MeCN containing 0.1% TFA over 15 min. After lyophilization, 10.8 mg (0.70 µmol, 71%) of the desired peptide were obtained as TFA salt.

HRMS-ESI$^+$ (m/z): $[M+18H]^{18+}$ calcd.: 707.1501; found: 707.1519.

2.13 Fmoc-Gly-D-Ser(psiMe,Mepro)-OH

Fmoc-Gly-D-Ser(psiMe,Mepro)-OH was synthesized according to the procedure of J. K. Clegg, *et al.* from Fmoc-Gly-D-Ser-OBz as starting materials.[3] Lyophilisation from *t*-BuOH yielded a white powder (55%).

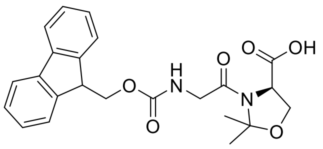

Fig. S14: Structure of Fmoc-Gly-D-Ser(psiMe,Mepro)-OH.

^{1}H NMR (500 MHz, CD_3CN)

δ 7.82 (d, *J* = 7.6 Hz, 2H), 7.67 (d, *J* = 7.5 Hz, 2H), 7.43 – 7.39 (m, 2H), 7.33 (td, *J* = 7.5, 1.2 Hz, 2H), 5.92 (NH, br, 4.56), (d, *J* = 6.2 Hz, 1H), 4.32 (d, *J* = 6.6 Hz, 2H), 4.28 – 4.21 (m, 2H), 4.16 – 4.11 (m, 1H), 3.91 (dd, *J* = 16.9, 5.5 Hz, 1H), 3.70 (dd, *J* = 16.9, 5.3 Hz, 1H), 1.59 (s, 3H), 1.49 (s, 3H)

^{13}C NMR (126 MHz, CD_3CN)

δ 172.22, 167.14, 157.49, 157.46, 145.12, 145.09, 142.09, 128.68, 128.11, 126.19, 126.17, 120.96, 118.33, 97.15, 67.38, 58.92, 47.96, 44.48, 44.38, 25.64, 23.49

HRMS-ESI^{+} (m/z): calcd. for $C_{23}H_{24}N_2O_6$ $[M+H]^+$: 425.1708; found: 425.1709.

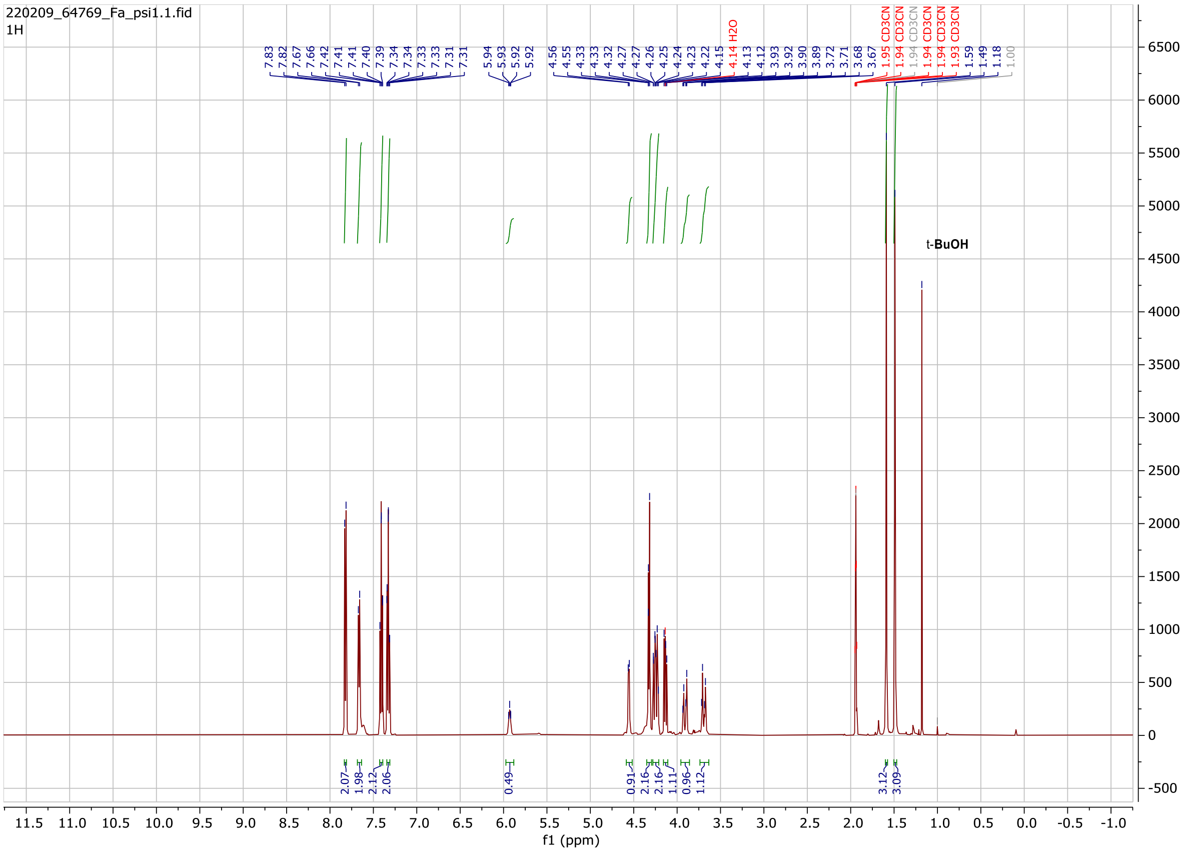

Fig. S15: ^{1}H NMR (500 MHz, CD_3CN) of Fmoc-Gly-D-Ser(psiMe,Mepro)-OH.

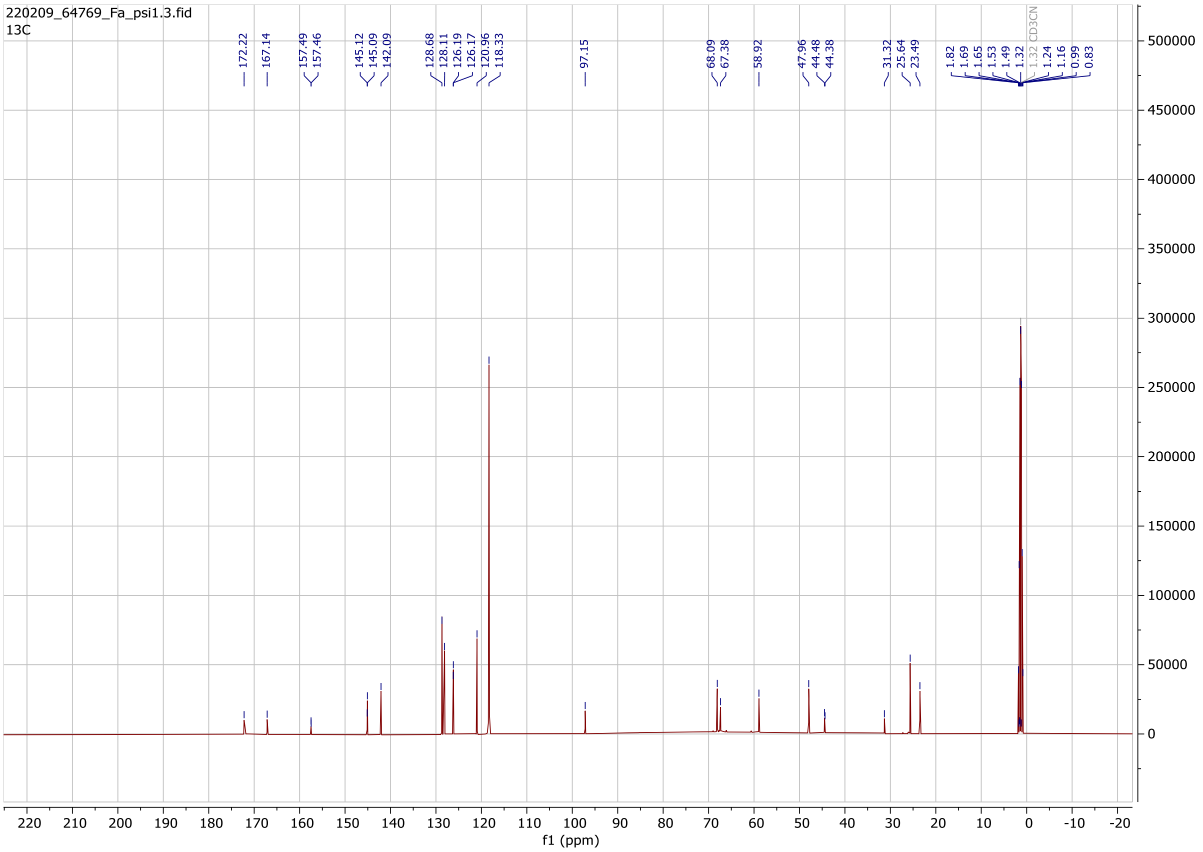

Fig. S16: ^{13}C NMR (126 MHz, CD_3CN) of Fmoc-Gly-D-Ser(psiMe,Mepro)-OH.

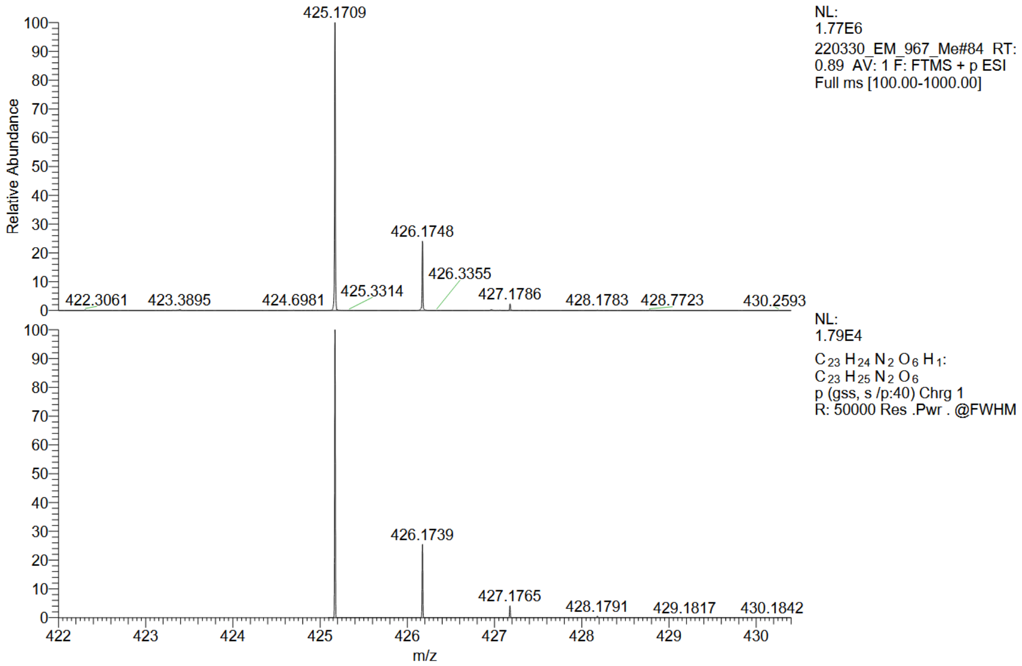

Fig. S17: HRMS-ESI$^+$ of Fmoc-Gly-D-Ser(psiMe,Mepro)-OH.

3 Refolding of synthetic Bcr-Abl SH2 (L-5 and D-5)

The lyophilized polypeptides were dissolved in solubilization buffer (6 M guanidine-HCl, 20 mM HEPES, pH 8.5, 150 mM NaCl, 0.5 mM TCEP-HCl) at a concentration of 0.5 mg/mL and transferred into a Slide-A-Lyzer G2 dialysis cassette with a cutoff of 3.5 kDa (Thermo Scientific). The solution was dialyzed against a 200-fold volume of refolding buffer (20 mM HEPES, pH 7.4, 150 mM NaCl, 0.5 mM TCEP-HCl) for 2 h at 4 °C. Then, the dialysis was repeated for 2 and 16 h by transferring the cassette into fresh refolding buffer. Afterwards, the solution containing the refolded protein was purified via size exclusion chromatography in refolding buffer on the Äkta Go system (Cytiva) with a HiLoad 16/600 Superdex 75 pg column to check for and remove aggregates at a flow rate of 1 mL/min. The fractions containing the refolded, synthetic Bcr-Abl SH2 domain were collected and concentrated up to 55.1 µM (0.7 mg/mL). The aliquots were flash frozen in liquid nitrogen and stored at -80 °C.

4 Expression and purification of recombinant Bcr-Abl SH2

Recombinant Bcr-Abl SH2 domain was expressed with an *N*-terminal tag containing His_6, GST and a tobacco etch virus (TEV) protease recognition motif using a modified pET expression vector. BL21(DE3) cells containing the plasmid were grown to OD_{600} = 0.8 at 37 °C in LB medium containing 50 µg/mL kanamycin. Afterwards, the cells were shifted to 18 °C and expression was induced via addition of IPTG at a final concentration of 0.5 mM. After 16 h, the cells were harvested by centrifugation at 6000 rpm and 4 °C for 15 min (Beckman Coulter JA-10 rotor, Beckman Coulter J2-MC centrifuge). The cells were resuspended in 30 mL of Ni-NTA buffer A (50 mM Tris-HCl, pH 7.5, 500 mM NaCl, 10 mM imidazole) per 1 L of bacterial culture containing EDTA-free protease inhibitor cocktail (Roche) and a spatula tip of DNase I (Roche) and lysed with an Avestin Emulsiflex C3 homogenizer (4 rounds, 10000 – 15000 psi). The suspension was centrifuged for 1 h at 4 °C and 15000 rpm (Beckman Coulter JA-17 rotor, Beckman Coulter J2-MC centrifuge) and the supernatant was filtered through a 5 µm filter (25 mm, Low Protein Binding Durapore Membrane, Merck Millipore). Protein purification was carried out using the *N*-terminal His_6 tag by nickel-affinity chromatography (column: 5 mL His-Trap FF crude, GE Healthcare) on an Äkta Avant system (GE Healthcare) according to the manufacturer's instructions. Bound proteins were eluted with a gradient from 10 to 500 mM imidazole over 10 column volumes. The His_6-GST tag was cleaved by addition of TEV protease in a 1:40 dilution to the collected protein fractions and incubation at 4 °C over night. The protein solution was further purified by size exclusion chromatography with buffer containing 20 mM HEPES, pH 7.4, 150 mM NaCl, 0.5 mM TCEP-HCl to remove the His_6-GST tag and TEV protease on the Äkta Avant system (column: HiLoad 16/600 Superdex 75 pg). The fractions were checked via SDS-PAGE and pooled. The protein was usually concentrated up to 610 µM (7.1 mg/mL), aliquoted, flash frozen in liquid nitrogen and stored at -80 °C. The obtained protein was verified by mass spectrometry.

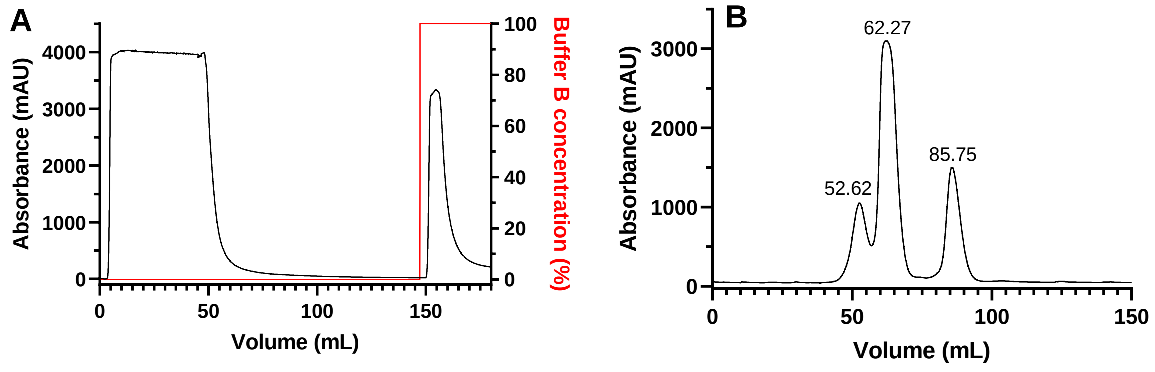

Fig. S18: A) Representative chromatogram of the Bcr-Abl SH2 purification via nickel-affinity chromatography measured at 280 nm; **B)** Representative chromatogram of the Bcr-Abl SH2 purification after TEV cleavage via size exclusion chromatography measured at 280 nm, the Bcr-Abl SH2 domain typically elutes around 86 mL. The peaks at ~62 mL and ~53 mL contain His_6-GST in different oligomerization states and TEV protease.

5 Expression and purification of recombinant His_{10}-Flag-AS25

The monobody AS25 was produced with an *N*-terminal tag containing His_{10} and FLAG as well as a tobacco etch virus (TEV) protease recognition motif in a modified pET expression vector. BL21(DE3) cells containing the plasmid were grown in autoinduction medium containing 50 µg/mL kanamycin at 37 °C. Upon reaching OD_{600} = 0.8, the cells were shifted to 18 °C. After 16 h, the cells were harvested by centrifugation at 6000 rpm and 4 °C for 15 min (Beckman Coulter JA-10 rotor, Beckman Coulter J2-MC centrifuge). The cells were resuspended in 30 mL of Ni-NTA buffer A (50 mM Tris-HCl, pH 7.5, 500 mM NaCl, 10 mM imidazole) per 1 L of bacterial culture containing EDTA-free protease inhibitor cocktail (Roche) and a spatula tip of DNase I (Roche) and lysed with an Avestin Emulsiflex C3 homogenizer (4 rounds, 10000 – 15000 psi). The suspension was centrifuged for 1 h at 4 °C and 15000 rpm (Beckman Coulter JA-17 rotor, Beckman Coulter J2-MC centrifuge) and the supernatant was filtered through a 5 µm filter (25 mm, Low Protein Binding Durapore Membrane, Merck Millipore). Protein purification was carried out on Ni-NTA Agarose beads (Qiagen) with 1 mL bead volume according to the manufacturer's instructions. The beads were washed with Ni-NTA buffer A (50 mM Tris-HCl, pH 7.5, 500 mM NaCl, 10 mM imidazole) and mixed with the supernatant containing His_{10}-Flag-AS25 for 30 min at 4 °C. The beads were centrifuged (2000 xg, 2 min, 4 °C), the supernatant was removed, and the beads were washed twice using Ni-NTA wash buffer (50 mM Tris-HCl, pH 7.5, 500 mM NaCl, 40 mM imidazole) with a centrifugation step (2000 xg, 2 min, 4 °C) in between. The bound protein was eluted with Ni-NTA buffer B (50 mM Tris-HCl, pH 7.5, 500 mM NaCl, 500 mM imidazole), the purity was checked by SDS-PAGE and the protein was dialyzed over night against a 200-fold excess volume of buffer containing 20 mM HEPES, pH 7.4, 150 mM NaCl, 0.5 mM TCEP-HCl in a Slide-A-Lyzer G2 dialysis cassette (cutoff 3.5 kDa, Thermo Scientific) at 4 °C. The protein was typically aliquoted in a concentration of ~107 µM (~1.5 mg/mL), flash frozen in liquid nitrogen and stored at -80 °C. The obtained protein was verified by mass spectrometry.

6 Isothermal titration calorimetry (ITC) measurements with His$_{10}$-Flag-AS25

Recombinant and synthetic proteins were dialyzed over night at 4 °C in ITC buffer (20 mM HEPES, pH 7.4, 150 mM NaCl, 0.5 mM TCEP-HCl). Protein concentration was measured on a NanoDrop 2000c (Thermo Scientific). ITC measurements were acquired on a MicroCal PEAQ-ITC instrument (Malvern Panalytical) and consisted in the titration of the monobody solution from the syringe in 19 steps with 0.4 µL for the first injection followed by 2.0 µL for the remaining steps with a spacing of 150 s between injections at 25 °C. The reference power, feedback and stir speed were set to 3.00 µcal/s, high and 750 rpm, respectively. Protein concentrations used were 200 µM of the monobody AS25 (syringe) titrated to a 20 µM solution of the recombinantly expressed or synthetic Bcr-Abl SH2 domain (cell). Thermodynamic parameters were determined with the MicroCal PEAQ-ITC analysis software.

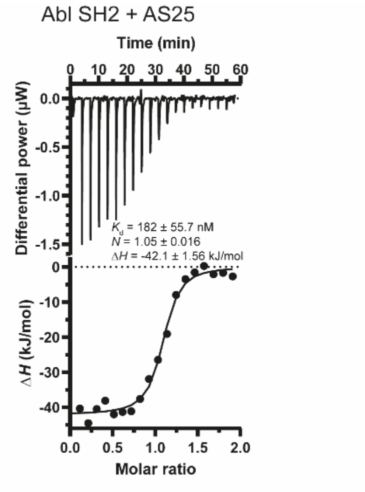

Fig. S19: ITC measurement of recombinantly expressed Bcr-Abl SH2 domain with its ligand AS25. All calorimetric titrations were performed at 25 °C. Each panel shows the raw heat signal of an ITC experiment (top) and the integrated calorimetric data of the area of each peak (bottom). The continuous line represents the best fit of the data based on a 1:1 binding model computed from the MicroCal software. A representative measurement of two independent experiments is shown with K_d value, stoichiometry (N) and enthalpy (ΔH) calculated from the fit. A list of all obtained K_d values can be found in Tab. S4. Here, AS25 (200 µM) is titrated to Abl SH2 (20 µM).

Tab. S4: Overview of the binding parameters of the interaction between recombinant or synthetic Bcr-Abl SH2 with the monobody AS25 obtained in two independent experiments.

	**1**	**2**
**Bcr-Abl SH2 + AS25**	K_d = 182 ± 55.7 nM<br>N = 1.05 ± 0.016<br>ΔH = -42.1 ± 1.56 kJ/mol	K_d = 219 ± 41.1 nM<br>N = 0.963 ± 0.010<br>ΔH = -29.0 ± 0.674 kJ/mol
**L-5 + AS25**	K_d = 184 ± 47.9 nM<br>N = 0.688 ± 0.011<br>ΔH = -26.7 ± 0.771 kJ/mol	K_d = 166 ± 33.5 nM<br>N = 0.449 ± 0.061<br>ΔH =-30.0 ± 0.688 kJ/mol

7 ITC measurements with phospho peptide pYEEI

Recombinant and synthetic proteins were dialyzed over night at 4 °C in ITC buffer (20 mM HEPES, pH 7.4, 150 mM NaCl, 0.5 mM TCEP-HCl). Protein concentration was measured on a NanoDrop 2000c (Thermo Scientific). Lyophilized pYEEI peptides in L- and D-configuration with the sequence 5-carboxyfluoresceine-EPQpYEEIPIYLK-$CONH_2$ were dissolved in the same buffer. ITC measurements were acquired on a MicroCal PEAQ-ITC (Malvern Panalytical) instrument and consisted in the titration of the peptide solution from the syringe in 13 steps with 0.4 µL for the first injection followed by 3.0 µL for the remaining steps with a spacing of 150 s between injections at 25 °C. The reference power, feedback and stir speed were set to 5.00 µcal/s, high and 750 rpm, respectively. 300 µM of peptide (syringe) were titrated to a 30 µM solution of the recombinantly expressed or synthetic Bcr-Abl SH2 domain (cell). Thermodynamic parameters were determined with the MicroCal PEAQ-ITC analysis software.

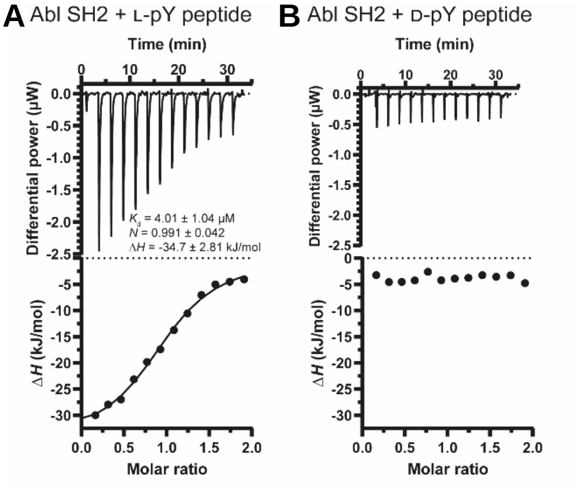

Fig. S20: ITC measurements of recombinantly expressed Bcr-Abl SH2 domain with its ligand, the pYEEI peptide. All calorimetric titrations were performed at 25 °C. Each panel shows the raw heat signal of an ITC experiment (top) and the integrated calorimetric data of the area of each peak (bottom). The continuous line represents the best fit of the data based on a 1:1 binding model computed from the MicroCal software. A representative measurement of two independent experiments is shown with K_d value, stoichiometry (N) and enthalpy (ΔH) calculated from the fit. A list of all obtained K_d values can be found in Tab. S5. **A)** L-pY peptide (300 µM) is titrated to Abl SH2 (30 µM); **B)** D-pY peptide (300 µM) is titrated to Abl SH2 (30 µM). As expected no binding was observed for B). The full sequence of the pY peptide is 5-Carboxyfluoresceine-EPQpYEEIPIYLK-CONH2.

Tab. S5: Overview of the binding parameters of the interaction between recombinant or synthetic Bcr-Abl SH2 with the phospho peptide pYEEI obtained in two independent experiments.

	**1**	**2**
**Bcr-Abl SH2 + L-pYEEI**	K_d = 4.01 ± 1.04 µM<br>N = 0.991 ± 0.042<br>ΔH = -34.7 ± 2.81 kJ/mol	K_d = 2.84 ± 1.05 µM<br>N = 1.11 ± 0.065<br>ΔH = -31.9 ± 3.44 kJ/mol
**L-5 + L-pYEEI**	K_d = 4.89 ± 1.07 µM<br>N = 0.604 ± 0.021<br>ΔH = -33.5 ± 02.80 kJ/mol	K_d = 4.94 ± 1.05 µM<br>N = 0.611 ± 0.020<br>ΔH =-33.8 ± 2.67 kJ/mol
**D-5 + D-pYEEI**	K_d = 4.18 ± 0.55 µM<br>N = 0.819 ± 0.016<br>ΔH = -36.7 ± 1.53 kJ/mol	K_d = 2.83 ± 1.26 µM<br>N = 0.667 ± 0.040<br>ΔH =-34.3 ± 4.31 kJ/mol

8 Circular dichroism (CD) measurements

The recombinant and synthetic proteins originally in HEPES buffer (20 mM HEPES, pH 7.4, 150 mM NaCl, 0.5 mM TCEP-HCl) were dialyzed three times against a 200-fold volume of phosphate-buffered saline (PBS) at pH 7.4, twice for 2 h and then for 16 h at 4 °C. Dialysis was carried out in Slide-A-Lyzer G2 dialysis cassettes (cutoff 3.5 kDa, Thermo Scientific). CD spectra were recorded in a quartz cuvette (path length: 0.1 cm, Hellma Analytics) containing 20 µg of sample in 300 µL PBS buffer (pH 7.4) on a JASCO J-815 circular dichroism spectrometer at 20 °C and a data interval of 0.1 nm. Samples were also measured in PBS buffer (pH 7.4) containing 6 M guanidine-HCl to obtain CD spectra of the unfolded proteins. The data was plotted using the software GraphPad Prism 8.

Tab. S6: Secondary structure content of recombinantly expressed and synthetic Bcr-Abl SH2 domains in L- and D-configuration. The percentages were calculated with BeStSel based on the obtained CD spectra from two independent measurements.[4]

Secondary structure element	Abl SH2	L-5	D-5
α-helix	7.20%	5.80%	4.00%
β-sheet	32.8%	26.1%	24.8%
β-turn	13.2%	15.6%	14.5%
Others	46.8%	52.4%	56.7%

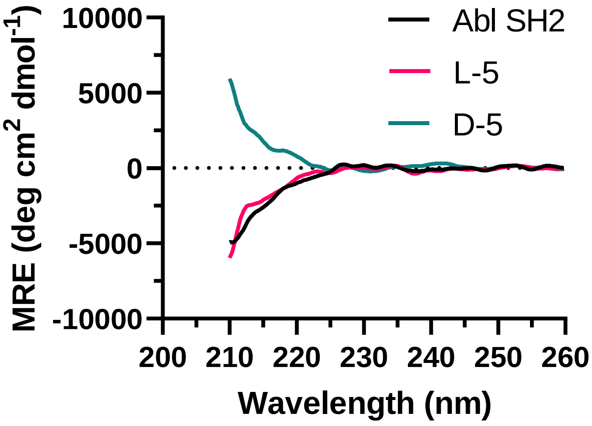

Fig. S21: Averaged far-UV circular dichroism (CD) spectra of recombinantly expressed and synthetic L- and D-Bcr-Abl SH2 domains, which were denatured in 6 M guanidine-HCl in PBS, pH 7.4. The mean residue ellipticity (MRE) was calculated according to T. E. Creighton.[5]

9 Nano differential scanning fluorimetry (nanoDSF) measurements

The thermal denaturation curves were determined by measurements of intrinsic tryptophan fluorescence. This analysis was performed using label-free, native differential scanning fluorimetry on a Prometheus NT.48 instrument (NanoTemper). Approximately 10 µL of the protein samples in PBS (pH 7.4) were loaded in Prometheus NT.48 capillaries. The tryptophan residues of the proteins were excited at 280 nm and the fluorescence intensity was recorded at 330 and 350 nm. Excitation power was set to 80 % and the temperature of the measurement compartment increased from 21 to 95 °C at a rate of 1 °C/min. Melting temperatures (T_m) were determined by the Prometheus software through calculation of the fluorescence ratio at 330 and 350 nm and of the first derivative. The data was plotted using the software GraphPad Prism 8.

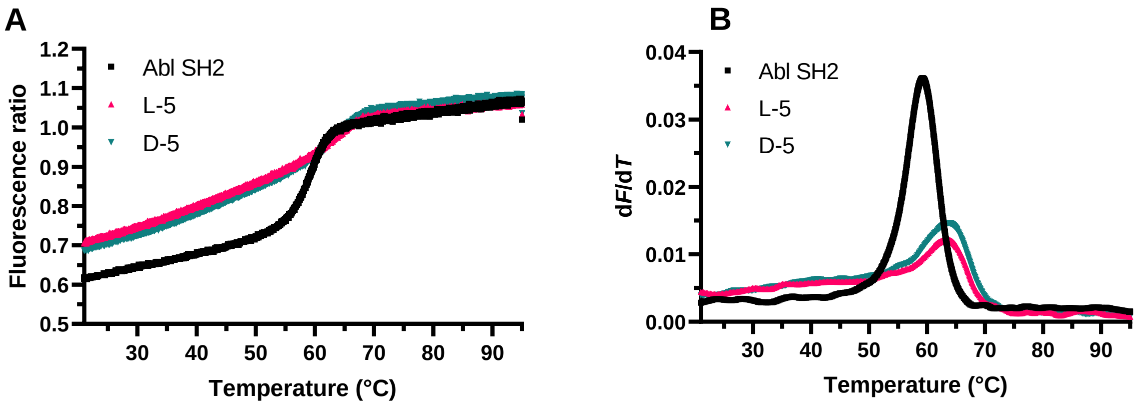

Fig. S22: A) Plot of the fluorescence ratio measured at 330 and 350 nm; **B)** Plot of the first derivative of the measured fluorescence ratio, the maximum indicates the melting temperature (T_m).

Tab. S7: Measured melting temperatures (T_m) from two independent experiments.

**Protein**	**T_m [°C], experiment 1**	**T_m [°C], experiment 2**
Bcr-Abl SH2	59.2	59.1
L-5	63.1	62.0
D-5	63.3	63.4

10 References

1. R. Winkler, *Rapid Commun. Mass Spectrom.*, 2010, **24**, 285-294.
2. K. M. Cergol, R. E. Thompson, L. R. Malins, P. Turner and R. J. Payne, *Org. Lett.*, 2014, **16**, 290-293.
3. J. K. Clegg, J. R. Cochrane, N. Sayyadi, D. Skropeta, P. Turner and K. A. Jolliffe, *Aust. J. Chem.*, 2009, **62**.
4. A. Micsonai, F. Wien, E. Bulyaki, J. Kun, E. Moussong, Y. H. Lee, Y. Goto, M. Refregiers and J. Kardos, *Nucleic Acids Res.*, 2018, **46**, W315-W322.
5. T. E. Creighton, *Protein structure : a practical approach*, IRL, Oxford, Repr. with corrections edn., 1990.
